# A new future for dog breeding

**DOI:** 10.1017/awf.2024.66

**Published:** 2025-01-13

**Authors:** Helle Friis Proschowsky, Maja Louise Arendt, Brenda N Bonnett, Camilla S Bruun, Irena Czycholl, Merete Fredholm, Dan O’Neill, James A Serpell, Peter Sandøe

**Affiliations:** 1 Department of Veterinary and Animal Sciences, University of Copenhagen, Grønnegårdsvej 8, DK-1870, Frederiksberg C, Denmark; 2 Department of Veterinary Clinical Sciences, University of Copenhagen, Dyrlægevej 16, DK-1870, Frederiksberg C, Denmark; 3 Department of Food and Resource Economics, University of Copenhagen, Rolighedsvej 23, DK-1958, Frederiksberg C, Denmark; 4 B Bonnett Consulting, Georgian Bluffs, ON, Canada; 5 Pathobiology and Population Sciences, The Royal Veterinary College, Hawkshead Lane, North Mymms, Hatfield, Herts AL9 7TA, UK; 6 School Veterinary Medicine, University of Pennsylvania, Philadelphia, PA 19104, USA

**Keywords:** Animal welfare, designer breed, extreme conformation, healthy breeding, mixed breed, purebred dog

## Abstract

The modern idea of purebred dogs has come under increasing critical scrutiny over recent decades. In light of this critical focus and other developments in society, some new trends in how companion dogs are bred and acquired have emerged. This means a diminishing influence from traditional kennel clubs with more dogs being sold without a pedigree, stricter legal restrictions on dog breeding, growing popularity of deliberate crosses of established breeds (i.e. so-called designer breeds) and growing hype around the benefits of mixed-breed dogs. We give an overview of these trends and discuss to what extent they will serve to promote dogs that are innately healthy, have good welfare and function well in their various roles in today’s world. We argue that newly invented designer breeds and mixed breeds also have worrying health and behavioural problems, and that the predictability of purebred dogs with respect to body size, basic behaviours, known need for grooming, disorder profiles and other attributes may well offer some benefits for a satisfying human-dog relationship seen from both sides. The optimal future seems to lie in the middle ground, where the future organised dog world (i.e. kennel and breed clubs or their successor organisations) will need to re-open the breed registries, remove wording from breed standards that currently promotes extreme conformation, support selection against disease-predisposing genotypes and phenotypes and refocus dog showing and breeding to promote health and appropriate behaviour.

## Introduction

The modern idea of purebred dogs has come under increasing critical scrutiny over recent decades, first by academics, and later in the public and the media. What is here referred to as purebred dog breeds are, as described later, the effects of man-made selection to create groups of dogs of similar appearance with a defined pedigree (a known recorded lineage), and their pedigreed or non-pedigreed descendants. A main concern has been that many dogs from high-profile breeds, despite many good intentions, end up suffering from a high burden of inherited disorders due to limited founder populations and inbreeding as well as disabilities resulting from deliberate selection for extreme conformation traits that negatively affect both their welfare and longevity (ICECDogs 2024). In light of this critical focus, some new trends in how companion dogs are bred and acquired have emerged in many countries across the globe. This means a diminishing influence from traditional kennel clubs with more purebred dogs sold without pedigrees, stricter legal restrictions on dog breeding (LAGECDogs 2024a), growing popularity of deliberate crosses of established breeds (i.e. so-called designer breeds) and growing hype around potential health benefits in mixed-breed dogs (O’Neill *et al.* 2023). In this Horizon paper, we aim to (a) give an overview of these trends, and (b) discuss to what extent they will serve to promote future dogs that are healthy, have good welfare and also function well in their various expected roles in today’s world.

## The rise and fall of the modern pedigree dog

### From the wolf to early breed-like types of dogs

The domestic dog originates from a close relative of today’s grey wolf through a domestication process that occurred between 40,000 and 15,000 years ago somewhere on the Eurasian continent and perhaps in more than one location (Frantz *et al.* 2016; Leeb 2023). Currently, there are two predominant “*origin stories*” describing this process (Serpell 2021).

The dominant story for many years has been the *commensal scavenger hypothesis*, proposing that wolves essentially domesticated themselves by staying close to Paleolithic human settlements in search of food scraps (Coppinger & Coppinger 2001). However, this hypothesis has recently been challenged by several arguments. One is that late Pleistocene humans were unlikely to have generated enough waste food to sustain a population of scavenging wolves (Jung & Pörtl 2018). Another counter-argument is that, for safety reasons, Pleistocene humans would have been unlikely to tolerate unsocialised wolves in such proximity to their settlements (Serpell 2021).

The alternative theory is known as *the pet-keeping* or *cross-species adoption hypothesis.* This theory derives legitimacy from observations of pet-keeping among contemporary societies of hunter-gatherers. According to this theory, habitual nurturing behaviour motivates humans to capture, adopt and rear infant mammals, such as wolf pups. During the Pleistocene epoch, most of these early pets would probably have reverted to living independently when they reached maturity, but a minority – those possessing socially desirable traits, such as tameability, trainability, and sociability – might have received favoured treatment and hence been more likely to remain under human control and survive to give rise to domestic descendants with similar characteristics (Serpell 2021).

Either way, selective pressures from the need to survive in a human environment have strongly shaped the modern dog’s genome and studies have identified genetic changes associated with dog domestication such as tameness, coat colour (Ostrander & Wayne 2005), and adaptation to a diet influenced by human food sources (Axelsson *et al.* 2013; Arendt *et al.* 2016).

Archaeological findings demonstrate that the relationship between humans and their dogs from these early days of domestication already extended beyond just the practical utility of dogs. Burials of dogs alongside humans 14,000 years ago in Germany (Janssens *et al.* 2018) and 12,000 years ago in Israel (Davis & Valla 1978) suggest that strong emotional connections existed between humans and dogs that are also documented in numerous other prehistorical findings and by modern ethnographic evidence (Morey 2006; Serpell 2017; pp 300–315). The Susa ceramics from 4,000 BC Iran seem to represent some of the oldest documentation of a recognisable breed type, with images of dogs that greatly resemble modern sighthounds, such as salukis and Afghan hounds (Hole & Wyllie 2007). Aligned with this, genetic studies have reported an ancient origin of sighthound breeds and other breed types (Parker *et al.* 2004; Dutrow *et al.* 2022).

### Development of modern dog breeding

While phenotypically diverse types of dogs – so-called blood lines – have existed for thousands of years, the modern notion of a ‘dog breed’ is a much more recent invention. This happened in Britain in the Victorian era in the second half of the 19th century. It was a time of great power and wealth for Britain, with expansion of the empire across the globe. The era was also characterised by industrial development, as well as rapid developments in science (Worboys *et al.* 2018).

The first documented dog show was held in Newcastle-upon-Tyne in 1859, the same year that Charles Darwin published *The Origin of Species.* The so-called dog fancy grew out of the earlier poultry and pigeon fancies, and the hobby breeding of prize-winning livestock that emerged in England during the late 18th and early 19th centuries (Ritvo 1987; pp 97–99). Consequently, breeding of dogs became closely related to dog-showing competitions and was linked to social status. As stated by the historian, Harriet Ritvo: *“The institutions that defined the dog fancy projected an obsessively detailed vision of a stratified order which sorted animals and, by implication, people into snug and appropriate niches”* (Ritvo 1987; p 93).

The foundation of the pedigree history of a breed was the stud book and later the breed registry. All members of a certain breed should either belong to a group of founder animals or should be offspring of these. In the beginning, the breed registry was open to allow entry of new founder animals with desirable phenotypes, but this practice ceased little-by-little. For each breed, an ideal conformation called a breed standard was described toward which breeders directed their selection efforts and based on which the judges at shows awarded their prizes (Skipper 2022a). Selection was required to maintain or improve the desired appearance, with ‘improve’ being taken to mean conforming more closely to these breed standards.

As already mentioned, some dog breeds formally recognised in the 1800s were preceded by breed-like types (or varieties or classes) of dogs. Earlier publications like Johannes Caius’s *De Canibus Britannicis* in Latin from 1570 and translated to English in 1576 described 17 varieties of dogs categorised by function and including names like ‘setters’ and ‘spaniels’ (Caius 1576). Despite bearing names prefiguring those of nineteenth-century breeds, these varieties were not necessarily similar in appearance to the modern dogs called setters or spaniels. It was not until the eighteenth century that the term ‘breed’ defined as *“a subspecies with definable physical characteristics that would reliably reproduce itself if its members were crossed with each other”* was invented, beginning with foxhounds (Ritvo 1987; p 93).

During the early years of the dog fancy, impartial and objective evaluation by show judges was an ideal that was hard to achieve in practice. Competition for prizes – some of which were pecuniary – was intense, and genuine fraud consequently took place behind the scenes of the dog shows. Coats of dogs were dyed to cover faults, ears and tails were surgically ‘corrected’, and false information was published in catalogues (Skipper 2020). To counteract the diminishing reputation of dog shows and dog breeding, after much political wrangling, a group describing themselves as “true sportsmen” formed the English Kennel Club – or just the Kennel Club (about to be renamed as the Royal Kennel Club) – in 1873. The main initial task of this new organisation was to combat fraud by establishing the identity and descent of pedigree dogs. At the same time, a system was developed that limited participation in dog shows, and thereby competition, to a carefully screened segment of the canine population (Ritvo 1987; p 102). From then – and continuing today – all pedigree dogs were required to have a recorded parentage documented in a breed registry. However, many breed registers were still open to non-pedigree individuals all the way into the 1950s and the overall registry of the Kennel Club was not officially closed until 1 January 1971 (Skipper 2022b).

Many other European countries followed this model. The French Societé Centrale Canine was established in 1882, and in Scandinavia, Sweden and Finland established national kennel clubs in 1889, Denmark in 1897 and Norway in 1898. The international umbrella organisation for dog breeding, the Fédération Cynologique Internationale (FCI), was founded in 1911 and includes kennel clubs from most of the world except the UK and US (Sandøe *et al.* 2015; p 15). The American Kennel Club was founded in 1884 by a group of American members of elite families who pledged “*to do everything to advance the study, breeding, exhibiting, running and maintenance of purity of thoroughbred dogs*” (Lemonick 2001).

The first studbook of the English Kennel Club identified 40 different dog breeds. This number has grown substantially over the years and in 2023, the year of the 150^th^ anniversary, more than 200 breeds were recognised (The Kennel Club 2024). Worldwide, around 400 different breeds are described by kennel clubs with over 800 breeds described by owners in the wider general public (O’Neill *et al.* 2023). Some of these breeds, as mentioned, resemble and derive from types of dogs that previously existed as geographic varieties (or landraces) used for hunting, herding, guarding and other functions. Others result from creative efforts of passionate and entrepreneurial individuals. The English golden retriever, for instance, was created by hybridising a yellow wavy-coated retriever over several generations with a spaniel, a setter, a Labrador retriever, and a bloodhound. Many modern breeds are, therefore, essentially early examples of what we currently call designer crossbreeding and represent the product of deliberate breeding involving a range of different breeds or types of dogs (Sampson & Binns 2006).

The adoption of structured pedigree dog breeding in combination with adoption of the English model of a national kennel club overseeing the process led to relatively uniform structures for dog fancier activities across Europe, North America, Australasia, and other parts of the world. Within individual countries, dog populations were divided into pedigree dogs and ‘the rest’. The founders of the kennel clubs articulated this division as a beneficial and necessary step towards promoting their contemporary concept of the healthier pure subset of the overall populations at a time where mainstream beliefs around dog health centred around upholding an aristocracy of ‘pure’ bloodlines and avoiding adulteration from inferior non-pedigree blood lines (Worboys *et al.* 2018). However, even then, some far-sighted individuals already predicted later problems related to inbreeding (Walsh 1879; p 265).

### Documentation and growing awareness of negative effects on health-related welfare of many breeds

More recent concerns regarding health disorders in purebred dogs have emerged gradually since the 1960s and are based on three dominant issues: (a) the unintended consequences of inbreeding; (b) selection for extreme conformation traits such as short muzzles (brachycephaly) and; (c) inadequate selection against disease-predisposing gene variants and phenotypes.

#### Inbreeding

The build-up of heritable diseases is a predictable result of increased homozygosity and progressive loss of genetic variation over time in closed breeding populations, no matter the species, due to selection and genetic drift. In dogs, however, some additional factors seem to have fueled the process. Many breeds were established with a limited number of founders so when the studbooks were closed – an event which occurred at variable times in different breeds and countries – the genetic variation was already small. Also, only a small proportion of each generation of dog breeds generally contributes to the next generation. Extensive use of popular sires (often dogs that have achieved extraordinary results at dog shows, utility tests or other kinds of dog sports) increases the frequency of the genetic variants they carry – including deleterious mutations – in the population. The widely promoted practice of deliberate inbreeding and line breeding – at least in the past – increased the risk of homozygosity for these variants in subsequent generations leading to the current situation with high levels of inherited disorders (Oliehoek *et al.* 2009; Leroy & Baumung 2011; Marsden *et al.* 2016; Broeckx 2020; Ikolo *et al.* 2023).

Some examples of these canine inherited disorders, include the eye disease ‘progressive rod-cone degeneration’ recorded in several breeds (Zangerl *et al.* 2006), disc herniation in dachshunds (Jensen & Christensen 2000) and the cardiac disease myxomatous mitral valve disease in cavalier King Charles spaniels (O’Brien *et al.* 2021). It should be noted that selection against disease-predisposing variants can result in homozygosity but of a desirable kind.

#### Breeding for extreme phenotypes

Extreme conformation in dogs describes a physical appearance that has been so significantly altered by selective breeding away from the ancestral natural canine appearance that affected dogs commonly suffer from poor health and welfare, with negative impacts on their quality and quantity of life (ICECDogs 2024). For example, the short muzzled (brachycephalic) breeds, such as French bulldog, English bulldog, and pug commonly have breathing difficulties, and dental, and eye problems (Packer *et al.* 2015; O’Neill *et al.* 2020; Packer & O’Neill 2022), the short, rounded skull of the cavalier King Charles spaniel and the Chihuahua can cause syringomyelia, a neurological disease, due to compression of the brain tissue (Chandler *et al.* 2008; Kiviranta *et al.* 2017), and excessive skin relative to the skeleton in several breeds increases the risk of skin-fold dermatitis (O’Neill et al. 2022a).

Within FCI, the breed standards, by which the show judges are required to evaluate dogs, are written and managed by the country of origin for each breed (FCI 2023). This also holds true for the British breeds even though the Kennel Club is not a member of FCI. In the US, the process is more decentralised, and the breed standards are written and maintained by the individual breed clubs (AKC 2024). The wording of some breed standards has unfortunately supported progressive exaggeration of certain breed characteristics over time. For example, the American Kennel Club breed standard for the English bulldog states that: “*The face, measured from the front of the cheekbone to the tip of the nose, should be extremely short”*, and later: “*The nose should be large, broad and black, its tip set back deeply between the eyes. The distance from the bottom of the stop, between the eyes, to the tip of the nose should be as short as possible and not exceed the length from the tip of the nose to the edge of underlip”* (AKC 2016). The equivalent standards from The Kennel Club and the FCI are slightly less extreme stating that the face of a bulldog should be “*relatively short, muzzle broad, blunt, and inclined slightly upwards, although not excessively so. Viewed from side, head appears very high and moderately short from back to point of nose”* (FCI 2011; KC 2023). Furthermore, these breed-defining phenotypes have tended to become exaggerated in some breeds over time – seldom because the wording of the breed standards has changed but more often because their interpretation by breeders and judges tends to drift.

Consequently, over the years, the muzzles of many dogs of brachycephalic breeds have become shorter, the ears of many basset hounds have become longer and the downward lumbar slope of the back of a German shepherd dog described in the standard as “slightly downwards” has over time become steeper in many dogs. The motivations behind these progressive exaggerations are intriguing but could be examples of what are termed *supernormal stimuli* in ethology (Tinbergen 1951). Here, exaggerated versions of a stimulus elicit a stronger response than the stimulus for which a specific behaviour evolved (Ghirlanda & Enquist 2003). In other words, when judging a group of quite uniform dogs, the one that stands out due to its more exaggerated features gets the most attention – and therefore wins the prize, thereby promoting even greater moves towards exaggeration in the future. This may also have affected across-breed comparisons which contribute to the final competitions for ‘Best in Show’ titles in dog shows (Markarian 2023). Regardless of cause, comparisons of historically old and new images of the same breed suggest that the exterior characteristics of many breeds have, and continue to, become more extreme over time (Sandøe *et al.* 2015; Serpell 2019).

#### Inadequate selection against disease-predisposing gene variants and phenotypes

All domestic animals have changed over time because of selection for different purposes. Today, livestock breeding is concentrated on relatively few, large international and commercial companies which operate within well-defined selection parameters related to yield, growth or other economically important traits. Dog breeding on the contrary is a decentral and often hobby-based activity, where breeding decisions are influenced by more individuals with far less objective selection criteria. Health-based breeding, or direct selection against disease-predisposing gene variants and phenotypes in specific breeds, has been prioritised to some extent (FCI 2020) but prize-winning at dog shows and other competitions as well as individual breeder’s preferences for type, and colour are also in play. The most common health-screening programmes include radiographic evaluations for hip and elbow dysplasia (FCI 2006; IEWG 2024), clinical eye examinations to detect inherited diseases like PRA (Progressive Retinal Atrophy) or cataract (ECVO 2024) and heart scans to screen for diseases like MMVD (Myxomatous Mitral Valve Disease) (Birkegård *et al.* 2016), DCM (Dilated Cardiomyopathy) or SAS (Sub-Aortal Stenosis) (ESVC 2024). An increasing number of DNA tests for inherited diseases have become available for dog breeders in recent years (IPFD 2024) and estimated breeding values, which have been a highly effective tool in livestock breeding, have also been introduced to a limited extent (Lewis *et al.* 2010). Some kennel clubs and breed clubs have breed-specific health criteria which must be met for puppies to be registered and assigned a pedigree, but the level of regulation and administrative practice for screening results varies greatly between countries and from breed-to-breed. In addition, this regulation only applies to the dogs within each breed which have a pedigree.

#### Growing public awareness of welfare problems linked to dog breeding

The BBC1 television documentary, *Pedigree Dogs Exposed*, aired in August 2008, is widely considered as the tipping point for public awareness and concern about many deleterious effects of organised dog breeding on dog health and welfare (Nicholas 2011; Lawler 2012). Similar concerns had been expressed and documented earlier but had failed to gain widespread public traction. One of the earliest investigations into physical defects in purebred dogs was carried out at the request of the Kennel Club in 1963 by the British Small Animal Veterinary Association (BSAVA) (Hodgman 1963). Many subsequent studies from a wide range of academic researchers (Peyer & Steiger 1998; McGreevy & Nicholas 1999; McGreevy 2007; Asher *et al.* 2009) addressed similar concerns. While veterinarians initially focused internationally on issues such as hip dysplasia in popular breeds of the day (e.g. German shepherd dogs and Labrador retrievers), concerns about brachycephaly were not prominent from the start, partly because at that time many of the seriously affected dog breeds were much less popular than they are today (Packer & O’Neill 2022; pp 127–151).

In parallel with growing concerns about health issues related to purebred dog breeding, the documentation of population-based evidence on key breed-related health and welfare issues also underwent rapid development based on improved access to reliable and sufficient clinical data (O′Neill *et al.* 2014; Gough *et al.* 2018). The development of systematic programmes of research that apply Big Data approaches with a specific focus on breed-related disorders now provide animal welfare scientists with sufficient evidence to support both calls for active change and the design of policy reforms. For example, the VetCompass^TM^ Research Programme at the Royal Veterinary College, with its stated focus on welfare issues of dogs since its inception in 2010, has generated over 100 publications on a wide range of topics related to canine breed health and welfare between 2012 and 2024 (VetCompass 2024). Unfortunately, to date, neither scientific evidence nor public awareness from media have proven overly effective in changing the dominant behaviours of many breed organisations or the public towards prioritising health over looks for popular breeds.

#### Slow and insufficient actions from kennel clubs

During the past 25 years, the national kennel clubs, and the international collaborations they belong to, have undertaken a number of initiatives, typically in the form of internal debates nationally and internationally and seminars for show judges, in efforts to respond to the major problems linked to the concept and practice of breeding pedigree dogs (Hedhammar & Indrebø 2011). Despite these well-intentioned initiatives, very little real-world change in the health, conformation, or welfare issues of problematic dog breeds appears to have been achieved.

As stated previously, many of today’s problems of pedigree dogs can be traced back to how these dog breeds were established, selected, and maintained through breeding in partially or fully closed populations or in the pursuit of exaggerated conformation traits. The way selective breeding is currently practiced for pedigree dog breeding generally results in progressively diminishing genetic diversity over time which impacts negatively on the health and welfare of the dogs (Leroy & Baumung 2011; Kraus *et al.* 2023). In efforts to redress this loss of diversity, some breed clubs have established outcrossing projects to attempt to rescue breeds with low genetic diversity and/or high incidence of genetic disease, such as the Norwegian lundehund (Melis *et al.* 2022; Powell 2011), and the Irish red and white setter (Irish Red and White Setter Club 2024). In 2023, the Swedish Kennel Klub approved a crossbreeding project for cavalier King Charles spaniel (SKC 2023) and the Finnish Kennel Club has done the same for both cavalier King Charles spaniel and French bulldogs (FKC 2023). As professor in veterinary neurology, Clare Rusbridge, stated in *The Guardian* in June 2024: “*Dog breeds must be “rebooted” through careful crossbreeding to save them from ingrained health problems*” (Davis 2024).

These outcrossing projects are usually time-limited and include a well-defined and limited number of dogs. To our knowledge, maintaining an open breed registry within the framework of a national kennel club is rare, but nevertheless exists. The Danish Swedish farmdog was reconstructed in the 1980s and received FCI-recognition in 2019. All the way through the reconstruction process, the breed register was kept open, and still remains open by the Scandinavian kennel clubs. This means that owners of dogs showing phenotypic resemblance with the Danish Swedish farmdog can have their dog evaluated by an authorised judge and – if approved – the dog is enrolled in the breed register with a blank pedigree (DSGK 2024). The Finnish kennel club has an open breed registry for a number of other breeds, including the Jack Russell terrier, Lapponian herder, Norbottenspitz, Pyrenean sheepdogs, and Finnish lapdog. Apart from the phenotypic evaluation, entry into the breed also requires a DNA profile and testing for breed-specific hereditary diseases (FKC 2024). As a way to obtain better health, the Danish kennel club decided to open the breed registry for the English bulldog, French bulldog and pug by 1 May 2024. Entry into the registry requires a phenotypic evaluation and a maximum grade for BOAS (Brachycephalic Obstructive Airway Syndrome) of 0 or 1 (DKC 2024a).

Some of the former utility breeds, such as the German shepherd dog, border collie and Labrador retriever, have become popular as show and family dogs. This has resulted in show lines where selection is based almost entirely on meeting the current preferences of dog show judges, and in lines of family dogs produced on a commercial basis to physically represent the common public perception of the breeds. In both cases, there has been a switch away from selecting for the original functional behavioural traits, such as hunting or herding ability, that once characterised these breeds. Consequently, separate ‘breeds within the breed’ have emerged consisting of utility (working) lines, show lines and family dogs that can show quite remarkable differences in phenotype as well as basic behaviours and activity levels (Duffy *et al.* 2008; Fadel *et al.* 2016). Often, there is practically no exchange of genetic material between the two groups resulting in stratification and reduced effective population sizes within each (Chang *et al.* 2009).

The likelihood of achieving dog owner’s satisfaction increases when the needs and expectations of the owner are compatible with the behaviour of the dog (Curb *et al.* 2013). Thus, mismatches between expectations of owners and the behaviours of dogs with respect to within-breed lines may increase the risk of a problematic dog-owner relationship.

The typical human behaviour of choosing breeding animals based on show champions and sometimes even because some influencers promote extreme variants of dogs, e.g. the ‘tea-cup’ Chihuahua (Redmalm 2014), has resulted in other serious issues. What Harriet Ritvo wrote about the first dog shows in the late 18^th^ century sadly still holds true: “*When judges selected the prizewinner, they were not simply recognizing a particular outstanding animal. At the same time, they were identifying the strain to which the prizewinner belonged as promising breeding material, and they were endorsing a type toward which other breeders should aspire*” (Ritvo 1987; p 101).

Even though many breed standards of the FCI and other kennel clubs have been revised numerous times since their first versions right up to the present day, and all FCI standards now contain the general phrase that “*any departure from the foregoing points should be considered a fault and the seriousness with which the fault should be regarded should be in exact proportion to its degree and its effect upon the health and welfare of the dog*”, there is still room for improvement of the health focus in the standards and in their application. The Nordic countries have formulated so-called “Breed Specific Instructions” (NKU 2023) in which judges at dog shows are informed about relevant health issues in selected breeds. However, despite all this, prizes are still awarded to dogs that show extreme conformation (Burke 2024). Sadly, therefore, limited progress in practice has been made based on the well-intentioned initiatives from the established breeding organisations.

#### Reactions from civil society and regulators

For decades, many Western countries concentrated their animal welfare policies and regulatory initiatives primarily on production and laboratory animals. Regulatory initiatives aimed at protecting the welfare of companion animals have been scarce, and there has been little legislation until recently specifically aimed at dogs over and above general formulations in some, but far from all, anti-cruelty and animal welfare statutes (Andersen *et al.* 2021). However, due to growing negative publicity and pressure from animal welfare non-governmental organisations (NGOs), governments and courts in some countries seem to have lost faith in the ability of the dog-breeding fraternity to reform itself from the inside. Consequently, national legislation is starting to be passed and/or more effectively enforced in a few countries (LAGECDogs 2024a).

In The Netherlands, the keeping and breeding of pets is regulated by Article 3.4 of the 2014 Dutch Animal Keepers Decree (Overheid 2024). According to this, the breeding of companion animals in ways that have negative effects on their welfare and health is prohibited. Hereditary defects and conformational traits with negative effects on welfare and health should be avoided as well as the passing of behavioural abnormalities to offspring. Animal protection organisations in The Netherlands have been particularly active in highlighting the impaired welfare of purebred dogs with extreme phenotypes, especially the brachycephalic breeds.

The Expertise Centre on the Genetics of Companion Animals at Utrecht University was asked by the Dutch authorities to provide guidelines for the enforcement of the decree and decided to focus initially on welfare issues of brachycephalic dogs. Based on knowledge about the health issues related to brachycephaly, a list of criteria was defined that all dogs used for breeding should comply with. The background and the criteria are described in a report commissioned by the Dutch Ministry of Agriculture, Nature and Food Quality in 2020 (van Hagen 2020).

As of May 2020, Dutch breeders are therefore required to have physical measurements carried out on their breeding dogs by a veterinary practitioner prior to breeding, following the criteria outlined by the ministry. A central, though debated, criterion is the calculation of a craniofacial ratio (CFR) which is the ratio between the length of the muzzle and the cranial length. The CFR should preferably be above 0.5. Based on the level of fulfilment of the criteria, a traffic light system is used to indicate whether the animals can be used for breeding (green), can be accepted during a transition period (yellow), or are unacceptable (red). However, very few individuals of some breeds can meet this yellow or green criterion (Packer *et al.* 2015; Liu *et al.* 2017) so in practice this amounts to a ban on breeding a number of flat-faced dog breeds. The legal actions in The Netherlands originally focused on breeders associated with the national kennel club, neglecting the fact that many individuals from the breeds in question are imported from outside the country in which the legislation had no effect. To address this issue, the Dutch Minister of Agriculture, Nature and Food Quality has announced that a general ban on the keeping of pets with “*harmful external characteristics*”, is being considered (FECAVA 2023).

An example of a legal action in relation to the welfare problems in purebred dogs comes from a lawsuit in Norway from 2020 to 2023. The Norwegian Society for Protection of Animals (NSPA) sued the Norwegian Kennel Club (NKC), the Norwegian Bulldog Club, the Norwegian Cavalier Club, three breeders of cavalier King Charles spaniels, and three breeders of English bulldogs for non-compliance with the Norwegian Animal Welfare Act § 25 which states that: “*breeding shall encourage characteristics resulting in robust animals that function well and have good health*”. The legislation goes on to specify that breeding cannot be carried out if it: “*reduces the animals’ ability to practice natural behaviour or gives rise to general ethical reactions*” (Lovdata 2009) (authors’ translation). The case was brought to the Oslo District Court, then to the Oslo Court of Appeal and finally to the Supreme Court which concluded that further breeding of cavalier King Charles spaniels with the current genetic pool is in violation of the Animal Welfare Act. The Supreme Court also ruled that English bulldogs must be bred under a breeding programme aimed at reducing the occurrence of diseases such as Brachycephalic Obstructive Airway Syndrome (BOAS) caused by extreme conformation (Supreme Court of Norway 2023).

Similarly, § 11b of the German Animal Welfare Act outlaws the breeding of animals where it is expected that, due to heredity, the animals themselves, or their offspring, will lack body parts or organs for appropriate use, or have body parts that are unsuitable, or reshaped in such a way that pain, suffering, or damage may occur as a result. It is also forbidden to breed animals if it is expected that the offspring will develop hereditary behaviour that may cause suffering, e.g. aggressive behaviour (Bundesministerium der Justiz 2023). In order to make the Animal Welfare Act in Germany clearer and more operational, an expert group appointed by the Federal Ministry of Food and Agriculture has produced the document “*Expert opinion on the interpretation of § 11b of the Animal Welfare Act*” (Bundesministerium für Ernährung und Landwirtschaft 2023). In addition, new, sharpened rules on dog shows came into force in Germany in January 2022. It is now prohibited for dogs to participate in shows and field trials if they have evidence of any of a range of hereditary conditions.

In England and Wales, the basis for the legal protection of dog health and welfare is provided by the Animal Welfare Act (AWA) (Legislation 2006). Legal safeguarding of dog welfare in England under the AWA was strengthened in 2018 when the government passed the Licensing of Activities Involving Animals (LAIA) Regulations in 2018 (Legislation 2018). The LAIA regulations govern a variety of activities involving animals, including that commercial dog breeders are legally required to apply for a licence from their local authority to breed dogs and, if successful, must abide by the provisions in the LAIA regulations. These state that “*no dog may be kept for breeding if it can reasonably be expected, on the basis of its genotype, phenotype or state of health, that the breeding from it could have a detrimental effect on its health or welfare or the health or welfare of its offspring*” (Legislation 2018). To this end, licence holders “*must take all reasonable steps*” to check that the dogs kept for breeding – male and female – have good physical and genetic health, good temperament, can see, breathe normally, are physically fit, can exercise freely, and “*must be aware of any health risks that may be specific to the particular type or breed of dog*” (GOV.UK 2024). Any person who fails to meet these criteria will be in breach of their licence and may face a penalty notice imposing an on-the-spot fine of up to £5,000; revocation of the licence; or in the case of a serious breach, court proceedings potentially resulting in an unlimited fine and a criminal record (LAGECDogs 2024b).

### Decreasing popularity of pedigree dogs

It is unknown how many dogs were excluded during the first years of the various national kennel clubs in countries where the breed registries were closed nor the size of the total population of dogs in those days. However, it is known that the total number of dogs in many countries and the distribution of individual breeds have fluctuated dramatically over time for many reasons, including times of economic boom and recession, changes in human demography and ‘fashion’ both at the national and international level (Herzog 2006). Current knowledge about the structure and numbers of the wider dog populations has also improved during the last 25 years for several reasons, including mandatory microchip marking, licensing, and registration of dogs in several countries and the advent of big data research programmes (O’Neill *et al.* 2023; McMillan *et al.* 2024).

For example, in Denmark, ID-marking and registration in the Danish Dog Registry (DDR) has been mandatory since 1993 (Retsinformation 2018). Analyses of data from the Danish Dog Registry and the registration figures provided by the Danish Kennel Club (DKC), show that the Danish dog population currently falls into three categories: (a) dogs with a pedigree from the DKC (around 35%); (b) dogs that are registered as a stated breed but without a DKC pedigree (around 50%); and (c) dogs that are registered as mixed breed (around 15%) (DKC 2024b).

These Danish proportional registrations of pedigree purebreds, purebreds without a pedigree, and mixed breeds are similar to those reported for the UK (O’Neill *et al.* 2023; KC 2024) and Germany (TASSO 2023) even though the legal basis and registration procedures for dogs differ between the countries. In Germany, microchip identification of dogs is regulated locally in the 16 different regions (‘Bundesländer’). In the UK, it became compulsory for owners to ensure that their dog is microchipped in 2016. However, several different microchip databases are in use that are not inter-linked, and there is no single overall UK national dog registry. In Scandinavia, Sweden introduced legislation like Denmark’s in 2001, but it is not possible to distinguish breeds to the same level of precision (Jordbruksverket 2001). Raad van Beheer, the kennel club of The Netherlands, has extended even beyond a requirement for microchipping with mandatory genetic profiling or ‘DNA fingerprinting’ (Raad van Beheer 2024).

**Figure 1.**
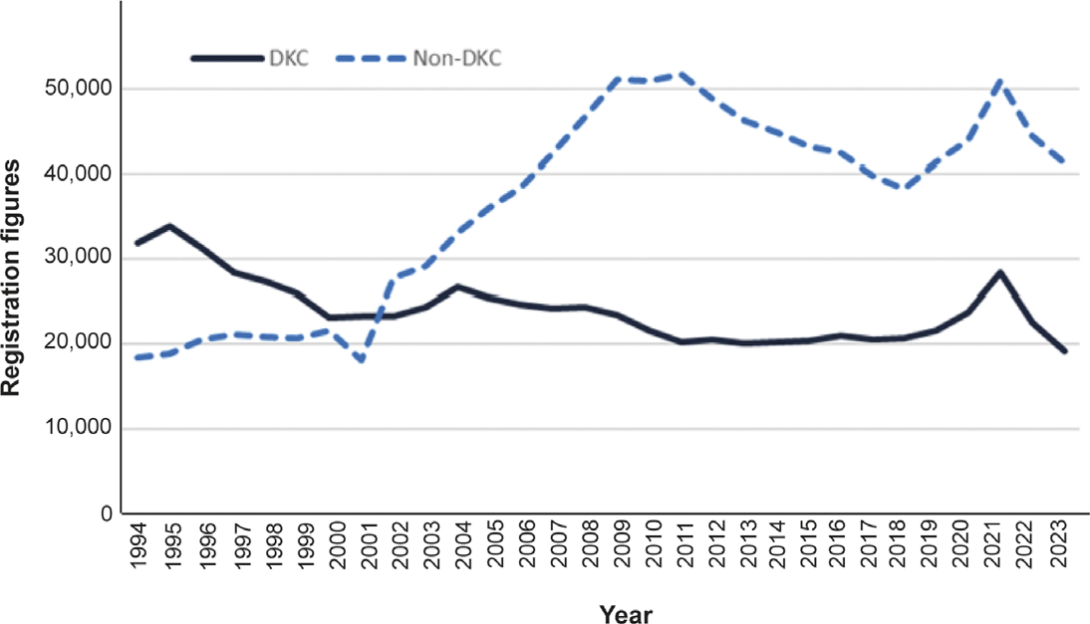
Registration figures from the Danish Dog Registry from 1994–2023. The dark blue, solid line represents the registration figures from the Danish Kennel Club (DKC), the light blue, dotted line represents the number of dogs entering the Danish Dog Registry without a pedigree including mixed breeds (non-DKC). The DKC proportion of all dogs has decreased from 63.5% in 1994 to 31.8% in 2023.

Data from the Danish Dog Registry reveal that the proportion of the Danish dog population that is kennel club registered has been falling over time. When mandatory microchipping was first enforced in 1993, the Danish Kennel Club (DKC) registered two-thirds of the total number of new puppies entering the register. This proportion decreased progressively over the following years until a cross-over point in 2002 where 51,035 new puppies were registered, 23,359 with a DKC pedigree (45.8%) and 27,676 without a DKC pedigree (54.2%) (Danish Dog Registry 2024; DKC 2024b; Figure 1).

In parallel with strong economic growth in the first years of the current millennium, demand for dogs as domestic pets in Denmark also increased. However, this demand was met mainly by suppliers from outside the DKC (Sandøe *et al.* 2022). Inability or even reluctance among the DKC breeders to scale-up dog production to meet increasing demand offers a possible explanation for this development. Globalisation of all markets for products and production, including that for puppies, and increasing sales from international online platforms that offer cheaper puppies and more convenient sales processes could be another. The financial crisis and subsequent recession in 2008 resulted in a plateau and then reduction in the annual number of dog registrations in Denmark. From 2018, the number of registrations increased again, a development which further accelerated during the global COVID-19 pandemic (Figure 1).

The popularity of different breeds also fluctuates over time. Urbanisation, changes in family constellations, and influence from movies and television series, social media, and celebrity dog owners along with many other factors contribute to a constant reshaping of breed ownership levels (Ghirlanda *et al.* 2013, 2014). In the past decade, many countries have experienced a shift in the top five most common breeds, away from larger breeds such as German shepherd dogs and Labrador retrievers, towards smaller breeds such as the Chihuahua and Havanese, and brachycephalic breeds such as the pug and French bulldog (KC 2019; Haid 2023; TASSO 2023).

**Figure 2.**
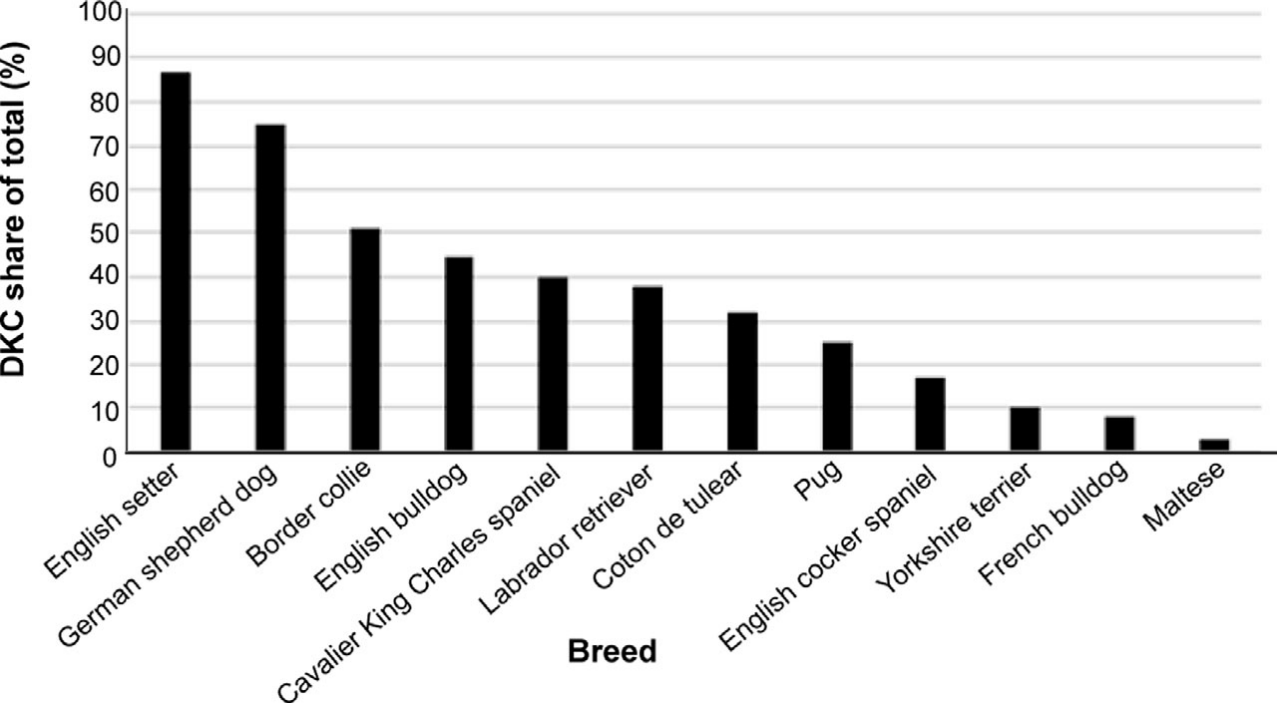
The percentage of dogs from different breeds entering the Danish Dog Registry in 2023 who had a pedigree from the Danish Kennel Club. Utility breeds originally designed for hunting and sporting show high proportional kennel club registration while many breeds that were originally popularised as companion animals show low levels of kennel club registration.

There are also differences when it comes to the relative number of dogs with a pedigree across the spectrum of breeds. In the Danish Dog Registry, utility breeds, originally selected and used for hunting, sporting and other physical activities and competitions, tend to include a high pedigree proportion maybe because the pedigree is a prerequisite for participation in some activities and competitions. The proportion of individuals with a DKC pedigree is lower in breeds that are largely kept as companion animals (Figure 2). Thus, the link between the DKC and the ownership of many of the popular companion breeds has reduced dramatically. Presumably, this situation is similar across other countries and kennel clubs and implies that the kennel clubs are losing control of several breeds where most of the breeding is taking place outside traditional dog-breeding organisations.

## Developments outside traditional dog breeding organisations

Besides declining proportions of purebred dogs who have a pedigree, several other developments in the last two to three decades have also affected how people in large parts of the world perceive and categorise ‘breed’ in dogs. So-called designer breeds and various other kinds of mixed breed dogs have become prominent contributors to widespread changes in dog-breed structures.

### Designer breeds

#### History and development of designer breeds

The emergence of the so-called ‘designer dog breeds’ (also known as ‘designer-crossbred dogs’) has provided a new twist to the concept of ‘breed’. Designer dog breeds are deliberate crosses between two or more different pure breeds and have gained great popularity over the past two decades. A study of the demography of 2.25 million UK dogs in 2019 within the VetCompass™ Programme estimated that designer crossbreeds made up 6.7% of the UK dog population, with poodle heritage being very common in these dogs (O’Neill *et al.* 2023).

Wally Conron, who was then working at the Royal Guide Dogs Association of Australia, is often credited with the invention of the first of these modern designer breeds, the labradoodle, in the 1980s. He crossed one of his best performing Labrador retrievers with a poodle to create a guide dog to help a blind woman whose husband was allergic to dogs. His approach appeared to be successful in terms of a reduced allergic reaction from the husband and this led to an assumption that still prevails widely today that the non-shedding fur of the poodle – in purebreds as well as in F1-hybrids – could reduce or prevent allergic reactions (Burnett *et al.* 2022).

This assumption of hypo-allergenicity has since been shown to be more complex than originally thought (Nicholas *et al.* 2011; Vredegoor *et al.* 2012) but is nevertheless often repeated in sales advertisements for many designer breeds that include poodle parentage. As well as the original labradoodle, these include cockapoo (cocker spaniel × poodle), cavapoo (cavalier King Charles spaniel × poodle), schnoodle (schnauzer × poodle) and maltipoo (Maltese × poodle). Mr Conron later expressed his regrets about triggering the whole phenomenon of designer breeds because the new trend encouraged unscrupulous people to set up puppy mills (Coren 2014). Unfortunately, once Pandora’s box had been opened, the list of creative combinations kept getting longer with ‘breeds’ such as puggle (pug × beagle), pomsky (Pomeranian × husky), chorkie (Chihuahua × Yorkshire terrier) and many others being added.

Interviews with owners of designer dogs suggest that they expect their dogs to have stable temperaments, be healthier than other dogs, and have hypoallergenic and non-shedding coats. Also, these owners have the overall perception that their designer dogs somehow fit better into domestic life compared to other dogs (Power 2012; Powell *et al.* 2018).

According to a recent study comparing owner expectations and reality associated with owning purebred dogs, mixed breeds, and designer breeds, owners generally appear to be happy with their choice of dog regardless of category. However, one area where the designer dogs apparently do not meet their owner’s expectations concerns the need for grooming. Among owners of ‘doodles’ (offspring resulting from the intentional mix of at least two purebreds, one of which was either a miniature, toy, or standard poodle), 18.2% lamented the cost and frequency of grooming required while the corresponding figures were lower for owners of mixed breeds (3.1%) and purebreds (5.2%) (Hladky-Krage & Hoffman 2022). That study also reported the decision to acquire a designer breed had been driven largely by the appearance of the breeds and by a perception that these dogs are healthier than purebred dogs (Hladky-Krage & Hoffman 2022). The popularity of designer breeds can thus to some extent be interpreted as a backlash to the perceived poor health condition of the traditional pedigree breeds. However, the validity of this perception of improved health in cross-bred dogs is still uncertain. The overall health and behaviour of designer crossbreeds may be no more than a regression to the mean of the values for these attributes in the parental breeds, as was suggested to explain the largely equivalent disorder risks overall between general crossbred dogs compared to purebred dogs in the UK (O′Neill *et al.* 2014).

#### Are designer breeds healthier than purebred dogs?

The general expectation of improved health in crossbreeds compared to purebred dogs has its roots in the concept of *hybrid vigour* or *heterosis.* This is the extent to which the average performance of first-cross (F1) individuals for a specified trait is superior to the average performance of their parental strains/breeds for that same trait (Nicholas 2010). Charles Darwin was the first scientist to examine the phenomenon in a systematic manner (Darwin 1859), and the existence of a strong hybrid vigour effect is a foundational assumption underlying many livestock breeding schemes. However, there is only limited evidence supporting a substantial hybrid vigour effect in dogs (Nicholas *et al.* 2016).

It is important to note that hybrid vigour is not a fixed overall effect but relies on factors including the presence, absence and frequency of disease-liability variants, the heritability of the specific traits, and the levels of inbreeding in the breeds that are crossed (Nicholas *et al.* 2016). Besides the general effects of hybrid vigour from a genetic diversity perspective, some designer breeds were also created in efforts to reduce the negative effects of extreme conformation of one or more of the parent breeds. For example, an expected increase in nose length on average in puggles (pug × beagle) compared to purebred pugs might be predicted to lead to reduced prevalence of BOAS although – to our knowledge – no systematic studies to confirm these effects are published. However, studies have shown that crossing two breeds with reported predisposition to obesity could lead to even higher levels of obesity in the hybrid dog, stressing that hybrid vigour is not a solution to all types of problems (Pegram *et al.* 2021; O’Neill et al. 2022b).

As designer breeds become more established in the general dog population, it is likely that future designer puppies will be produced increasingly from backcrosses to one of the parental breeds or of intercrosses between F1 and later generations of designer crossbred parents (Arendt *et al.* 2024). This will turn designer breeds into *de facto* new breeds where, for example, all the parents, grandparents, great-grandparents and so forth of labradoodle pups will also have been labradoodles. This is now the case in Switzerland where the Swiss Kennel Club accepts labradoodles as a registered breed (Swiss Cynological Federation 2024). Therefore, any positive effects of heterosis are likely to wear out over time, and in terms of breeding-related health problems the designer breeds are likely to face similar problems to those faced by traditional breeds unless steps are taken to avoid the issues of inbreeding, the breeding towards extreme conformation and the lack of adequate focus on reducing disease-predisposing gene variants that have plagued so many of the longer-established dog breeds.

As with the alluring simplicity but faulty logic of considering all pedigree dogs as one group, it is also dangerous to consider all designer breeds as being a single entity, given the range of health risks and issues that could affect their various progenitor breeds (Gough *et al.* 2018). The limited research that does exist comparing mental and physical health in designer breeds to pure breeds has delivered mixed results. For example, a prospective study of ophthalmic disorders in labradoodles compared with their parental breeds showed that the prevalence of multifocal retinal dysplasia (MRD) was significantly higher in labradoodles than in Labrador retrievers and poodles. For cataracts, no significant differences were identified (Nicholas *et al.* 2016).

The standard poodle is recognised as genetically predisposed to hypoadrenocorticism (Friedenberg *et al.* 2017). A UK VetCompass^TM^ study reported the standard poodle with 51.38 times the odds of hypoadrenocorticism compared to general crossbred dogs while the Labrador retriever had 0.82 times the odds. However, the poodle-based designer crossbreed labradoodle showed an intermediate risk between the two progenitor breeds rather than a total elimination of the combined parental predisposition, with 7.40 times the odds (Schofield *et al.* 2021). New diseases, which have not been passed down from the parental breeds, have also emerged in designer breeds. For instance, a novel mutation in the dystrophin gene causing a disorder similar to human Duchenne muscular dystrophy (DMD) has been identified in a line of Australian labradoodles (Shrader *et al.* 2018).

A recent study from the UK compared the odds for 57 common disorders across three designer-crossbreeds (cavapoo, cockapoo, and labradoodle) and each of their progenitor breeds (cavalier King Charles spaniel, cocker spaniel, Labrador retriever, and poodle). The odds did not differ significantly between the designer-crossbreeds and their relevant progenitor breeds in 86.6% of the comparisons challenging the widespread beliefs of major positive hybrid vigour effects for health in designer-crossbreeds (Bryson *et al.* 2024).

A special concern for designer-crossbreeds is their mental health and the resultant expressed behaviours. Selective breeding over the past century has created a range of traditional dog breeds which differ in behaviour and personality traits. The degree of genetic attribution to both working behaviours, including herding and retrieving, and other everyday behaviours such as sociability, fearfulness, and aggression is debated, but it is widely accepted that behaviour is to some extent heritable (Saetre *et al.* 2006; MacLean *et al.* 2019; Dutrow *et al.* 2022). Still, very little is known about the expression of behavioural traits in crossbreeds including designer-crossbreeds compared to the behaviour of the constituent pure breeds.

Based on quantitative genetics theory, there are good reasons to anticipate that first-cross designer dogs would exhibit behavioural phenotypes closely aligned with an intermediate profile between their purebred parent breeds (Falconer & Mackay 2009 p 254). This expectation generally held true with some notable exceptions in a study of goldendoodles and labradoodles (Shouldice *et al.* 2019). The study analysed breed differences using the online Canine Behavioural Assessment and Research Questionnaire (C-BARQ; Hsu & Serpell 2003). Labrador retriever and standard poodle did not differ significantly from those of labradoodles for any behaviour category, but miniature poodles scored significantly higher than labradoodles for dog rivalry (aggressive or threatening responses to other familiar dogs in the same household). The goldendoodle had higher scores than the parent breeds for both dog-directed aggression and dog-directed fear. To our knowledge, no studies have analysed whether specific combinations between breeds are more likely to result in problematic behaviour than others, but this is a question of key relevance for future research given that most designer-crossbreds are destined for the companion animal market.

Though few studies have so far been published on the health of designer breeds, it seems likely that the assumption of a substantial general health benefit compared to their progenitor breeds is overestimated. Paradoxically, unless the new designer dog breeds are recognised or registered by the national kennel clubs or some other canine organisations, their health will not be systematically monitored, and they will remain outside of health programmes currently used by the organised dog world. However, coming under the umbrella of national kennel clubs could increase these health issues over time in designer breeds as a result of inbreeding, absence of selection against disease predisposing phenotypes and genetic variants and exaggeration towards extreme conformation, just as has happened in many of the traditional breeds.

### Mixed-breed dogs with unknown genetic background

#### Health and longevity

Prior to the emergence of designer breeds, a mixed breed would typically have been the result of casual or unintentional mating between dogs of different breeds or even dogs that were themselves already mixed breeds. Such mixed-breed dogs remain common in most general dog populations. A study of over 2 million dogs under UK veterinary care in 2019 revealed that approximately every fourth dog (24%) was classified as non-designer-crossbred (O’Neill *et al.* 2023). The equivalent figure from the Danish Dog Registry is 15% (Danish Dog Registry 2024).

As with designer breeds, dogs that are non-designer, mixed breed continue to have their advocates, and it is often the potential health benefits from crossbreeding that are highlighted. Several studies have analysed differences in general health between purebred and mixed-breed dogs. In England, data from electronic patient records were used to compare the prevalence of common disorders between purebred and mixed-breed dogs attending primary-care veterinary practices (O′Neill *et al.* 2014). Among the twenty most-frequently recorded disorders, purebred dogs had a significantly higher prevalence compared with crossbreds for otitis externa, obesity, and skin lumps. Overall, the study concluded that purebreds showed significantly higher prevalence values for 13 of the 84 (15.5%) disorders but substantial variation was shown across the pure breeds so that it was hard to disentangle the effects of pure breeding *per se* from the effects of being one of the many individual pure breeds. No instances were identified in which disorder prevalence values were significantly higher in mixed breeds than in purebred dogs overall.

A study from the University of California-Davis evaluated electronic records of referral patients from the Veterinary Medical Teaching Hospital for selected disease categories, including cancer, cardiac disorders, endocrine disorders, and orthopaedic disorders (Bellumori *et al.* 2013). From a total of 24 disorders assessed, 13 did not show a significant difference in risk between purebred and mixed-breed dogs after matching the two populations for age, sex, and bodyweight. Ten disorders were more prevalent in purebred dogs compared with mixed-breed dogs, including aortic stenosis, dilated cardiomyopathy, hypothyroidism, elbow dysplasia and intervertebral disc disease. Mixed-breed dogs were, on the other hand, more prone to cranial cruciate ligament rupture (CCLR) and trauma from car accidents than purebred dogs. Since the latter injuries would be likely to reflect differences in levels of physical exercise and human supervision, it is probable that their higher prevalence in mixed-breed dogs indicates more *laissez faire* owner attitudes rather than any innate predispositions of the dogs, although breed-related predisposition to CCLR and putative genetic components have been demonstrated (Baird *et al.* 2014).

A study of owner-reported survey data collected through the Dog Aging Project (DAP) Health and Life Experience Survey for 27,541 companion dogs concluded that, although individual breeds may show higher lifetime prevalence for specific conditions, purebred dogs did not show higher lifetime prevalence of medical conditions compared to mixed-breed dogs, and a higher proportion of purebred dogs than mixed-breed dogs had no owner-reported medical conditions (Forsyth *et al.* 2023).

Recent developments in DNA technology have made it possible to look beyond just clinical health and to study specific disease variants implicated in Mendelian disorders. A recent Finnish study screened around one million dogs for 250 genetic variants across 150 countries and reported that 87.9% of the variants were found in both purebred and mixed-breed dogs (Donner *et al.* 2023). Mixed-breed dogs were more likely to be heterozygote carriers of common recessive diseases, whereas purebreds were more likely to be genetically affected (homozygous) but 57% of all dogs in the study carried at least one copy of a known disease-associated variant. The study was a follow-up from a similar survey in 2018 which concluded that the allele frequencies for the most frequent disease variants are essentially the same in purebred and mixed-breed dogs, emphasising their common genetic background and shared inherited disease variants (Donner *et al.* 2018).

Average longevity is often used as a proxy scale for overall health, and several studies have reported on longevity for different breeds and mixed breeds. Research on dog longevity has primarily reported average ages at death based on data from veterinary practice (Fleming *et al.* 2011; O’Neill *et al.* 2013), owner questionnaires (Proschowsky *et al.* 2003; Lewis *et al.* 2018), health insurance databases (Bonnett & Egenvall 2010) or combined data sources (McMillan *et al.* 2024).

In most studies, the average longevity differs significantly between breeds. Mixed breeds usually end up at the higher end of the spectrum but are surpassed by some purebreds such as terriers and poodles (Proschowsky *et al.* 2003; Lewis *et al.* 2018). A recent study applied a different approach and calculated life tables for 30,563 dogs in the UK (Teng *et al.* 2022). Life expectancy tables express the remaining average life expectancy from different ages and provide more information than just the average age at death (i.e. average longevity) across all ages. At age 0, the life expectancy for all dogs in the dataset was 11.23, but life expectancy tables varied from 12.72 years in Jack Russell terriers to 4.53 years in French bulldogs. The overall category of mixed breeds had a life expectancy of 11.82 at age 0. A study based on combined data from breed registries, veterinary corporations, pet insurance companies, animal welfare charities, and academic institutions found that in comparison with the crossbred group, 47.1% of the pure breeds presented longer median survival estimates, 25.8% presented shorter, and 27.1% did not vary significantly from the crossbred group (McMillan *et al.* 2024).

Dogs belonging to large-bodied breeds are reported to generally have shorter average lifespans than dogs from physically smaller breeds (Galis *et al.* 2007; Greer *et al.* 2007). A negative correlation between longevity and body size was demonstrated in a study of 44,363 dogs representing 134 breeds. However, very small dogs seemed to have a reduced lifespan indicating that the correlation is non-linear and may reflect the effects of extreme conformation at either end of the dog size scale (Galis *et al.* 2007). This shortened life effect on extremely small breeds was also demonstrated in the life expectancy table study in which breeds such as the Chihuahua had a life expectancy at age 0 of only 7.91 (Teng *et al.* 2022).

Studies of lifespan and longevity, like the ones presented above, usually evaluate mixed breeds as a single group in the breed-based analyses. The main reason for this can be lack of data regarding size, bodyweight or the progenitor breeds constituting the individual mixed-breed dogs. It is, however, excessively simplistic to consider all mixed-breed dogs as one common group. For example, in many parts of Northern Europe, purebred dogs commonly comprise up to 70–80% of the overall population (O’Neill *et al.* 2023; TASSO 2023; DKC 2024b; KC 2024). Here, practically no truly outbred dogs exist, and most mixed-breed dogs are likely to have identifiable breeds among their closest ancestors.

The finding that some pure breeds outlived mixed breeds overall highlights the great variation between the longevities of the longest- and the shortest-lived pure breeds and suggests that the longevity of any one mixed-breed dog may be highly contingent on whether the parent breeds belonged to short- or long-lived breeds and on the disease-related cause of shortened longevity. Or, to put it in other words: there may be no such thing as *the* mixed breed but instead there are many different mixed breeds whose health and longevity will depend heavily on that of the (often unknown) progenitor breeds.

#### Behaviour in purebred and mixed-breed dogs – is the latter less predictable?

It is widely acknowledged that cohabitation between dogs and humans does not always run smoothly, and some dogs develop behaviours that owners may find problematic. Several studies using prevalence data have identified higher levels of reported problematic behaviours in mixed breeds compared to purebreds. Mixed-breed dogs are, for instance, reported to be more nervous, more excitable, and to exhibit excessive barking more frequently (Bennett *et al.* 2007), to be ranked higher for different kinds of aggression (Hsu & Sun 2010) and to be at increased risk of developing noise phobia (Blackwell *et al.* 2013).

Modern DNA technology can reveal insights into which breeds may have contributed to the genetic profile of an individual mixed breed dog and, despite debate about the accuracy of these tests, the Wisdom Panel homepage claims that their DNA test is able to explain: *“why your dog has to herd your whole family after dinner, loves chasing bunnies or hates baths*” (Wisdom Panel 2024). The implicit message here is that much of the behaviour expressed by dogs is determined by their breed affiliation. This argument is routinely used by the organised dog world to promote acquisition of a pedigree dog instead of a designer-crossbred or mixed breed or even a purebred without a pedigree. However, although the genetic basis for physical traits like body size (Sutter *et al.* 2007) and fur type (Cadieu *et al.* 2009) is largely unchallenged, the extent to which behaviour is determined by genetics continues to be debated.

A much-publicised 2022 study questioned the hereditary basis of dog behaviour by exploring correlations between breed characteristics and DNA-sequencing results from more than 2,000 purebred and mixed-breed dogs (Morrill *et al.* 2022). The authors reported that even though most behavioural traits are heritable to some extent, behaviour itself could not reliably differentiate between breeds, with breed itself explaining only 9% of variation in behaviour. So, contrary to the widespread beliefs about the importance of choosing a dog breed based on breed-related behaviours that will fit your needs and everyday circumstances, this study concluded that “*dog breed is generally a poor predictor of individual behaviour and should not be used to inform decisions relating to selection of a pet dog*”. The authors claim that the modern domestic dog is a recent invention defined by arbitrary rules regarding physical appearance and purity of ‘bloodlines’ rather than behaviour and that this modern breeding approach is widely detached from the pre-Victorian lineages of dogs that were selected for functional roles such as hunting, guarding, and herding that were largely predicated on predictable behaviours in these dogs.

A contradictory conclusion was drawn in a paper by Dutrow *et al.* (2022), which identified genetic drivers of canine behaviour using data from more than 4,000 domestic, semi-feral and wild dogs, and phenotypic data from over 46,000 purebred dogs based on the Canine Behavioural Assessment and Research Questionnaire (C-BARQ; Hsu & Serpell 2003). The authors concluded that diversification of canine behavioural phenotypes predates the formation of modern breeds and identified ten major canine lineages each of which is associated with a unique repertoire of behavioural characteristics related to their historical functional roles (e.g. hunting, herding, scent trailing, etc) reflected in many modern breeds. A Finnish study examined environmental and demographic factors associated with seven personality traits in a survey of over 11,000 dogs and concluded that “*a dog’s breed is not a predictor of its personality, but the probability of showing certain personality traits differs between breeds.*” Thus, the breed of the dog was the most important determinant underlying personality differences (Salonen *et al.* 2023).

A questionnaire study of 7,700 purebred dogs representing more than 200 breeds, and 7,691 mixed-breed dogs living in Germany reported that, according to their owners, mixed breeds were less calm, less sociable toward other dogs, and showed more problematic behaviour than purebreds (Turcsán *et al.* 2017). Arguing that factors like early socialisation and rearing environment may substantially impact canine behaviour, the authors re-analysed the dataset controlling for the distribution of the demographic and dog-housing factors and concluded that the lower sociability of mixed-breed dogs towards other dogs seemed to be an indirect result of the environmental differences between purebreds and mixed breeds.

The differing inferences from these various studies may reflect differences in the methods used to measure behaviour across breeds and breed types. In addition, most studies also acknowledge substantial within-breed variation when it comes to behaviour. Further research using robust measures of behaviour will be needed to determine the extent to which ‘breed’ is a reliable indicator of expected personality in purebred dogs compared with mixed breeds.

## A new future for dog breeding?

The current strong evidence on the scale of reduced health and welfare linked to breed as a concept in dogs and specifically driven by reduced genetic diversity, absence of health-conscious selection against disease predisposing phenotypes and genetic variants, and breeding towards extreme conformation means that actions to redress these issues are urgently needed. This Horizon paper should be taken as both a clarion call for action as well as offering some options for meaningful change. At a time where ideals such as ‘diversity’, ‘naturalness’, and ‘respect for ethnic diversity’ are trending in the human world, it can be tempting to call for a knee-jerk shutting down of all kennel and breed clubs and for humanity to turn our backs on a system of organised dog breeding that has been failing dogs in many ways for over a century.

However, such a draconian approach is unlikely to solve the health and welfare problems of dogs. Newly invented designer breeds and mixed breeds are shown to have important health and behavioural problems too, and the predictability of purebred dogs to some degree with respect to body size, basic behaviours, known need for grooming, disorder profiles and other attributes may well offer some benefits for a mutually fulfilling human-dog relationship.

Some critics of purebred dogs demand an end to breeding and recommend that prospective dog owners rescue dogs from shelters either at home or abroad – the ‘Adopt, Don’t Shop’ mantra (Hanson 2016; Adopt Don’t Shop 2024). However, in many countries, rescue dogs can only satisfy a small fraction of the wider public demand for dogs. A study in Denmark found that around 2,000 dogs enter shelters each year while the annual number of dogs acquired in Denmark is over 60,000 (Sandøe *et al.* 2019). This figure might look different in other countries, but the widespread keeping of dogs as pets means that in most places dogs will have to be bred in large numbers. So, stopping breeding is not the answer (Mäki 2023).

The way forward in terms of improving the breeding-related welfare of companion dogs seems to lie in the middle ground, where whatever future organised dog world exists then (i.e. updated versions of current kennel and breed clubs that have the health of dogs at their core or whatever organisations take their place) would move from the continuing obsession with prioritising the physical appearance of dogs to instead prioritising the overall health and welfare of the dogs under its remit. This would be in line with some current movement by some national kennel clubs and international organisations to publicly acknowledge that past processes prioritising closed breeding pools and promoting breed standards that encouraged extreme conformation no longer fit with the canine welfare understanding and demands of modern human society (FCI 2020). Such a new world would offer more transparent and evidence-based breeding systems that would reverse inbreeding effects and enforce a practice of healthy breeding with direct selection against disease-predisposing phenotypes and gene variants.

Retaining a system of organised dog breeding with formal registers into the future, in our opinion, offers two major advantages compared to uncontrolled dog breeding: traceability and transparency. For pedigree dogs, each puppy can be traced back to the parents and to the breeder who was responsible for producing the litter. Registration of breeding animals and their progeny in a central database of an organisation such as a kennel club – or even in international databases – enables more effective monitoring of results from health and breeding schemes, number of litters, levels of inbreeding etc, through several generations in a more transparent way.

Some countries have decided to focus on legislation as their primary tool to enforce human actions aimed at improving canine health and welfare. The design of these legislative actions currently differs widely between the countries. As much of the current legislation that can be applied to dog health and dog breeding is relatively new and these efforts to actively enforce legislative approaches are relatively recent, there is as yet insufficient evidence to determine the overall effectiveness of this legal approach to trigger positive and widespread change. Hopefully, reliable evaluation will emerge in the years to come to show some benefits to dog welfare. A major advantage from most forms of legislative action is that they, at least theoretically, apply to the entire population of dogs and not simply to pedigree dogs which is the case with breed-specific health schemes administered by the kennel clubs. Hitherto, a specific challenge has been the increasing importation of puppies from countries without regulation of canine breeding and welfare, but this can be expected to change when new EU legislation comes into force. According to a proposal from the EU Commission, imports will be subject to the same or equivalent standards (European Council 2024).

Most production animal breeding programmes today are based on genetic markers linked to desired traits – so-called genomic selection (Meuwissen *et al.* 2016) and selective breeding has clearly shown the potential for improvements in production and fertility traits with low heritability such as litter size in pigs (Rodenburg *et al.* 2003; Knap & Su 2008). In addition, digital tools have been shown to perform better than human observers when it comes to, for example, reliability of the evaluation of health and welfare in cattle and pigs (Benjamin & Yik 2020; Chapa *et al.* 2020; Bortoluzzi *et al.* 2023). Even though these tools may seem promising and theoretically have the power to be applied in dog breeding as well, several obstacles must be overcome before realistic implementation becomes possible for the breeding of companion dogs. These include, among others, definition, and agreement on evaluation parameters – some of which may differ from breed-to-breed – and registration of uniform, high quality phenotypes together with obtained genotype data.

## Animal welfare implications

The challenge remains to breed dogs with better physical and mental health. In our opinion, it is now time for those currently in charge of organised dog breeding to take responsibility for this challenge and to put the health and welfare of the dogs ahead of human goals such as heeding tradition, profit, winning prizes at shows and other outcomes that often currently are at odds with the well-being of the dogs themselves. Amongst others, such changes to longstanding practices that are now sadly recognised to be harmful to canine health and welfare could include re-opening the breed registries, removing, or modifying wording from breed standards that currently promotes extreme conformation, maintaining a high effective population size and promoting diversity rather than uniformity within breeds or breed-like dog types. The changes need to be drastic and if those currently charged with responsibility for organised dog breeding feel unable to rise to this challenge, then perhaps it is time that they should step aside and let others who *do* prioritise the health and welfare of dogs to take the reins. Health and welfare rather than looks should become the new goal in dog breeding. In addition, the responsibility to adhere to breed-, breed type- or conformation-specific phenotypic and genotypic selection criteria for good health outcomes must be clear for every person who produces puppies whether they are purebreds, mixed breeds or designer breeds.

## References

[r1] Adopt Don’t Shop 2024 *Adopt don’t shop – rehoming neglected animals.* https://adoptdontshop.eu/ (accessed 15 May 2024).

[r2] AKC 2016 *Bulldog Dog Breed Information.* https://www.akc.org/dog-breeds/bulldog/ (accessed 22 April 2024).

[r3] AKC 2024 *American Kennel Club.* https://www.akc.org/ (accessed 29 January 2024).

[r4] Andersen SS, Meyer I, Forkman B, Nielsen SS and Sandøe P 2021 Regulating companion dog welfare: A comparative study of legal frameworks in western countries. Animals 11(6): 1660. 10.3390/ani11061660 34199669 PMC8228344

[r5] Arendt M, Cairns KM, Ballard JWO, Savolainen P and Axelsson E 2016 Diet adaptation in dog reflects spread of prehistoric agriculture. Heredity 117(5): 301–306. 10.1038/hdy.2016.48 27406651 PMC5061917

[r6] Arendt ML, Fagerlund TM, Gelskov LV, Lorenz SAN, Nielsen SS and Sandøe P 2024 Hvorfor er labradoodles så populære, og hvad består de af? Resultater fra en dansk spørgeskemaundersøgelse. *Dansk Veterinærtidsskrift* 4. https://dvt.ddd.dk/bladarkiv/2024/nr-4/hvorfor-er-labradoodles-saa-populaere-og-hvad-bestaar-de-af-resultater-fra-en-dansk-spoergeskemaundersoegelse/ (accessed 13 November 2024). [Title translation: Why are labradoodles so popular and what are they made of? Results of a Danish questionnaire].

[r7] Asher L, Diesel G, Summers JF, McGreevy PD and Collins LM 2009 Inherited defects in pedigree dogs. Part 1: Disorders related to breed standards. Veterinary Journal 182(3): 402–411. 10.1016/j.tvjl.2009.08.033 19836981

[r8] Axelsson E, Ratnakumar A, Arendt M-L, Maqbool K, Webster MT, Perloski M, Liberg O, Arnemo JM, Hedhammar Å and Lindblad-Toh K 2013 The genomic signature of dog domestication reveals adaptation to a starch-rich diet. Nature 495(7441): 360–364. 10.1038/nature11837 23354050

[r9] Baird AEG, Carter SD, Innes JF, Ollier WE and Short AD 2014 Genetic basis of cranial cruciate ligament rupture (CCLR) in dogs. Connective Tissue Research 55(4): 275–281. 10.3109/03008207.2014.910199 24684544

[r10] Bellumori TP, Famula TR, Bannasch DL, Belanger JM and Oberbauer AM 2013 Prevalence of inherited disorders among mixed-breed and purebred dogs: 27,254 cases (1995–2010). Journal of the American Veterinary Medical Association 242(11): 1549–1555. 10.2460/javma.242.11.1549 23683021

[r11] Benjamin M and Yik S 2020 Precision livestock farming and technology in swine welfare. In: Benjamin E and Yik S (eds) Improving Animal Welfare: A Practical Approach, *3rd Edition* pp 376–384. CABI: Wallingford, UK.

[r402] Bennett PC, Cooper N, Rohlf VI and Mornement K 2007 Factors Influencing Owner Satisfaction With Companion-Dog-Training Facilities. Journal of Applied Animal Welfare Science 10(3), 217–241. 10.1080/10888700701353626 17645407

[r12] Birkegård AC, Reimann MJ, Martinussen T, Häggström J, Pedersen HD and Olsen LH 2016 Breeding restrictions decrease the prevalence of myxomatous mitral valve disease in Cavalier King Charles spaniels over an 8‐ to 10‐year period. Journal of Veterinary Internal Medicine 30(1): 63–68. 10.1111/jvim.13663 26578464 PMC4913653

[r13] Blackwell EJ, Bradshaw JWS and Casey RA 2013 Fear responses to noises in domestic dogs: Prevalence, risk factors and co-occurrence with other fear related behaviour. Applied Animal Behaviour Science 145(1–2): 15–25. 10.1016/j.applanim.2012.12.004

[r401] Bonnett BN and Egenvall A 2010 Age Patterns of Disease and Death in Insured Swedish Dogs, Cats and Horses. Journal of Comparative Pathology 142, S33–S38. 10.1016/j.jcpa.2009.10.008 19932895

[r14] Bortoluzzi EM, Goering MJ, Ochoa SJ, Holliday AJ, Mumm JM, Nelson CE, Wu H, Mote BE, Psota ET, Schmidt TB, Jaberi-Douraki M and Hulbert LE 2023 Evaluation of precision livestock technology and human scoring of nursery pigs in a controlled immune challenge experiment. Animals 13(2): 246. 10.3390/ani13020246 36670787 PMC9854951

[r15] Broeckx BJG 2020 The dog 2.0: Lessons learned from the past. Theriogenology 150: 20–26. 10.1016/j.theriogenology.2020.01.043 32000992

[r16] Bryson GT, O’Neill DG, Brand CL, Belshaw Z and Packer RMA 2024 The doodle dilemma: How the physical health of ‘Designer-crossbreed’ Cockapoo, Labradoodle and Cavapoo dogs’ compares to their purebred progenitor breeds. PLoS One 19(8): e0306350. 10.1371/journal.pone.0306350 39196904 PMC11355567

[r17] Bundesministerium der Justiz 2023 *TierSchG - Tierschutzgesetz.* https://www.gesetze-im-internet.de/tierschg/BJNR012770972.html (accessed 15 May 2024). [Title translation: Animal Protection Act].

[r18] Bundesministerium für Ernährung und Landwirtschaft 2023 *Gutachten zur Auslegung von Paragraf 11b des Tierschutzgesetzes.* https://www.bmel.de/DE/themen/tiere/tierschutz/gutachten-paragraf11b.html (accessed 13 December 2023). [Title translation: Report on the interpretation of section 11b of the Animal Welfare Act].

[r19] Burke O 2024 *Crufts viewers hit out at ‘appalling’ competition after RSPCA make statement.* https://www.ladbible.com/entertainment/tv/crufts-2024-controversy-rspca-french-bulldog-elton-611562-20240309 (accessed 15 May 2024).

[r20] Burnett E, Brand CL, O’Neill DG, Pegram CL, Belshaw Z, Stevens KB and Packer RMA 2022 How much is that doodle in the window? Exploring motivations and behaviours of UK owners acquiring designer crossbreed dogs (2019-2020). Canine Medicine and Genetics 9(1): 8. 10.1186/s40575-022-00120-x 35610665 PMC9127489

[r21] Cadieu E, Neff MW, Quignon P, Walsh K, Chase K, Parker HG, VonHoldt BM, Rhue A, Boyko A, Byers A, Wong A, Mosher DS, Elkahloun AG, Spady TC, André C, Lark KG, Cargill M, Bustamante CD, Wayne RK and Ostrander EA (2009) Coat Variation in the Domestic Dog Is Governed by Variants in Three Genes. Science 326(5949), 150–153. 10.1126/science.1177808 19713490 PMC2897713

[r168] Caius J 1576 Of Englishe dogges. Theatrum Orbis Terrarum; Da Capo Press: Amsterdam, New York.

[r22] Chandler K, Volk H, Rusbridge C and Jeffery N 2008 Syringomyelia in cavalier king Charles spaniels. The Veterinary Record 162(10): 324. 10.1136/vr.162.10.324-a 18326849

[r23] Chang ML, Yokoyama JS, Branson N, Dyer DJ, Hitte C, Overall KL and Hamilton SP 2009 Intrabreed stratification related to divergent selection regimes in purebred dogs may affect the interpretation of genetic association studies. Journal of Heredity 100: S28–S36. 10.1093/jhered/esp012

[r24] Chapa JM, Maschat K, Iwersen M, Baumgartner J and Drillich M 2020 Accelerometer systems as tools for health and welfare assessment in cattle and pigs – A review. Behavioural Processes 181: 104262. 10.1016/j.beproc.2020.104262 33049377

[r25] Coppinger R and Coppinger L 2001 Dogs: a startling new understanding of canine origin, behaviour, and evolution. Scribner: New York, USA.

[r26] Coren S 2014 A designer dog-maker regrets his creation. https://www.psychologytoday.com/us/blog/canine-corner/201404/designer-dog-maker-regrets-his-creation (accessed 14 December 2023).

[r27] Curb LA, Abramson CI, Grice JW and Kennison SM 2013 The relationship between personality match and pet satisfaction among dog owners. Anthrozoös 26(3): 395–404. 10.2752/175303713X13697429463673

[r28] Danish Dog Registry 2024 Nøgletal, antal hunde registreret. https://www.hunderegister.dk/om-os/n%C3%B8gletal (accessed 29 January 2024). [Title translation: Key figure, number of dogs registered].

[r29] Darwin C 1859 On the Origin of Species by Means of Natural Selection; Or, The Preservation of Favored Races in the Struggle for Life. John Murray: London, UK.

[r30] Davis N 2024 Dog breeds must be ‘rebooted’ to halt health problems, says expert. *The Guardian 2 June.* https://www.theguardian.com/lifeandstyle/article/2024/jun/02/dog-breeds-must-be-rebooted-to-halt-health-problems-says-expert-has-said?CMP=oth_b-aplnews_d-1 (accessed 13 November 2024).

[r31] Davis SJ and Valla FR 1978 Evidence for domestication of the dog 12,000 years ago in the Natufian of Israel. Nature 276(5688): 608–610. 10.1038/276608a0

[r32] DKC 2024a *Opening of breed registers for Frensh bulldog, English bulldog and pug.* https://www.dkk.dk/nyheder/2024/forbedring-af-sundhed-for-fransk-bulldog-engelsk-bulldog-og-mops (accessed 5 August 2024).

[r33] DKC 2024b *Yearly registrations in the Danish Kennel Club.* https://www.dkk.dk/om-dkk/love-instrukser-og-blanketter/årlige-registreringstal (accessed 29 January 2024)

[r34] Donner J, Anderson H, Davison S, Hughes AM, Bouirmane J, Lindqvist J, Lytle KM, Ganesan B, Ottka C, Ruotanen P, Kaukonen M, Forman OP, Fretwell N, Cole CA and Lohi H 2018 Frequency and distribution of 152 genetic disease variants in over 100,000 mixed breed and purebred dogs. PLoS Genetics 14(4): e1007361. 10.1371/journal.pgen.1007361 29708978 PMC5945203

[r35] Donner J, Freyer J, Davison S, Anderson H, Blades M, Honkanen L, Inman L, Brookhart-Knox CA, Louviere A, Forman OP and Chodroff Foran R 2023 Genetic prevalence and clinical relevance of canine Mendelian disease variants in over one million dogs. PLoS Genetics 19(2): e1010651. 10.1371/journal.pgen.1010651 36848397 PMC9997962

[r36] DSGK 2024 Dansk/Svensk Gårdhundeklub. https://www.dsgk.dk (accessed 1 February 2024). [Title translation: Danish/Swedish farm dog club].

[r37] Duffy DL, Hsu Y and Serpell JA 2008 Breed differences in canine aggression. Applied Animal Behavior Science 114(3-4): 441–460. 10.1016/j.applanim.2008.04.006

[r38] Dutrow EV, Serpell JA and Ostrander EA 2022 Domestic dog lineages reveal genetic drivers of behavioural diversification. Cell 185(25): 4737–4755. 10.1016/j.cell.2022.11.003 36493753 PMC10478034

[r39] ECVO 2024 *European College of Veterinary Ophthalmologists.* https://www.ecvo.eu/ (accessed 8 August 2024).

[r40] ESVC 2024 *European Society of Veterinary Cardiology.* https://www.esvcardio.org/ (accessed 8 August 2024).

[r41] European Council 2024 *Welfare of cats and dogs: Council paves the way for first ever EU-wide law.* https://www.consilium.europa.eu/en/press/press-releases/2024/06/26/welfare-of-cats-and-dogs-council-paves-the-way-for-first-ever-eu-wide-law/ (accessed 5 August 2024).

[r42] Falconer DS and Mackay T 2009 Introduction to quantitative genetics, *Fourth Edition.* Harlow: Pearson, Prentice Hall.

[r43] Fadel FR, Driscoll P, Pilot M, Wright H, Zulch H and Mills D 2016 Differences in trait impulsivity indicate diversification of dog breeds into working and show lines. Scientific Reports 6(1): 22162. 10.1038/srep22162 26963916 PMC4785826

[r44] FCI 2006 *FCI Requirements for Official Hip Dysplasia Screening.* https://www.fci.be/medias/SCI-REG-DYS-HAN-DIR-en-15518.pdf (accessed 8 August 2024).

[r45] FCI 2011 *FCI standard, Bulldog.* https://www.fci.be/en/nomenclature/BULLDOG-149.html (accessed 4 April 2024).

[r46] FCI 2020 *Healthy Breeding & Dog Welfare: informative package.* https://www.fci.be/en/Healthy-Breeding-Dog-Welfare-informative-package-3573.html (accessed 3 April 2024).

[r47] FCI 2023 *FCI Internal Rules.* https://www.fci.be/en/FCI-Internal-Rules-4774.html (accessed 22 April 2024).

[r48] FECAVA 2023 *The Netherlands is first country in the world to ban ownership of pets with harmful external characteristics.* https://www.fecava.org/boas/the-netherlands-is-first-country-in-the-world-to-ban-ownership-of-pets-with-harmful-external-characteristics/ (accessed 30 January 2024).

[r49] FKC 2023 *The Finnish Kennel Club accepted separate cross breeding projects for Cavalier King Charles Spaniels and French Bulldogs.* https://www.kennelliitto.fi/en/about-us/news/finnish-kennel-club-accepted-separate-cross-breeding-projects-cavalier-king-charles-spaniels-and-french-bulldogs (accessed 15 May 2024).

[r50] FKC 2024 *Programme for Combating Hereditary Diseases and Defects (PEVISA).* https://www.kennelliitto.fi/lomakkeet/pevisa-ja-rotukohtaiset-erityisehdot (accessed 5 August 2024).

[r51] Fleming JM, Creevy KE and Promislow DEL 2011 Mortality in North American dogs from 1984 to 2004: An investigation into age‐, size‐, and breed‐related causes of death. Journal of Veterinary Internal Medicine 25(2): 187–198. 10.1111/j.1939-1676.2011.0695.x 21352376

[r52] Forsyth KK, McCoy BM, Schmid SM, Promislow DEL, Snyder-Mackler N and the DAP Consortium 2023 Lifetime prevalence of owner-reported medical conditions in the 25 most common dog breeds in the Dog Aging Project pack. Frontiers in Veterinary Science 10: 1140417. 10.3389/fvets.2023.1140417 38026653 PMC10655140

[r53] Frantz LAF, Mullin VE, Pionnier-Capitan M, Lebrasseur O, Ollivier M, Perri A, Linderholm A, Mattiangeli V, Teasdale MD, Dimopoulos EA, Tresset A, Duffraisse M, McCormick F, Bartosiewicz L, Gál E, Nyerges ÉA, Sablin MV, Bréhard S, Mashkour M, Bălăşescu A, Gillet B, Hughes S, Chassaing O, Hitte C, Vigne J-D, Dobney K, Hänni C, Bradley DG and Larson G 2016 Genomic and archaeological evidence suggest a dual origin of domestic dogs. Science 352(6290): 1228–1231. 10.1126/science.aaf3161 27257259

[r54] Friedenberg SG, Lunn KF and Meurs KM 2017 Evaluation of the genetic basis of primary hypoadrenocorticism in Standard Poodles using SNP array genotyping and whole-genome sequencing. Mammalian Genome 28(1–2): 56–65. 10.1007/s00335-016-9671-6 27864587 PMC5495560

[r55] Galis F, Van Der Sluijs I, Van Dooren TJM, Metz JAJ and Nussbaumer M 2007 Do large dogs die young? Journal of Experimental Zoology Part B: Molecular and Developmental Evolution 308B(2): 119–126. 10.1002/jez.b.21116 16788896

[r56] Ghirlanda S, Acerbi A and Herzog H 2014 Dog movie stars and dog breed popularity: A case study in media influence on choice. PLoS One 9(9): e106565. 10.1371/journal.pone.0106565 25208271 PMC4160180

[r57] Ghirlanda S, Acerbi A, Herzog H and Serpell JA 2013 Fashion vs function in cultural evolution: The case of dog breed popularity. PLoS One 8(9): e74770. 10.1371/journal.pone.0074770 24040341 PMC3770587

[r58] Ghirlanda S and Enquist M 2003 A century of generalization. Animal Behaviour 66(1): 15–36. 10.1006/anbe.2003.2174

[r59] Gough A, Thomas A and O’Neill DG 2018 Breed Predispositions to Disease in Dogs and Cats, *Third Edition.* Wiley Blackwell: Hoboken, NJ, USA.

[r60] GOV.UK 2024 *Dog breeding licensing: statutory guidance for local authorities.* https://www.gov.uk/government/publications/animal-activities-licensing-guidance-for-local-authorities/dog-breeding-licensing-statutory-guidance-for-local-authorities (accessed 15 May 2024).

[r61] Greer KA, Canterberry SC and Murphy KE 2007 Statistical analysis regarding the effects of height and weight on life span of the domestic dog. Research in Veterinary Science 82(2): 208–214. 10.1016/j.rvsc.2006.06.005 16919689

[r62] Haid M 2023 *Most Popular Dog Breeds of 2022.* https://www.akc.org/expert-advice/dog-breeds/most-popular-dog-breeds-2022/ (accessed 13 December 2023).

[r63] Hanson M 2016 Stop buying pedigree dogs. Stop breeding them. Stop these awful practices. *The Guardian.* https://www.theguardian.com/commentisfree/2016/mar/15/pedigree-dogs-breeding-crufts-german-shepherd-best-in-breed (accessed 15 May 2024).

[r64] Hedhammar Å and Indrebø A 2011 Rules, regulations, strategies and activities within the Fédération Cynologique Internationale (FCI) to promote canine genetic health. The Veterinary Journal 189(2): 141–146. 10.1016/j.tvjl.2011.06.011 21757380

[r65] Herzog H 2006 Forty-two thousand and one dalmatians: Fads, social contagion, and dog breed popularity. Society & Animals 14(4): 383–397. 10.1163/156853006778882448

[r66] Hladky-Krage B and Hoffman CL 2022 Expectations versus reality of designer dog ownership in the United States. Animals 12(23): 3247. 10.3390/ani12233247 36496768 PMC9736103

[r67] Hodgman SFJ 1963 Abnormalities and defects in pedigree dogs–I. An investigation into the existence of abnormalities in pedigree dogs in the British Isles. Journal of Small Animal Practice 4(6): 447–456. 10.1111/j.1748-5827.1963.tb01301.x

[r68] Hole F and Wyllie C 2007 The oldest depictions of canines and a possible early breed of dog in Iran. Paléorient 33(1): 175–185. https://www.jstor.org/stable/41496803 (accessed 13 November 2024).

[r69] Hsu Y and Serpell JA 2003 Development and validation of a questionnaire for measuring behaviour and temperament traits in pet dogs. Journal of the American Veterinary Medical Association 223(9): 1293–1300. 10.2460/javma.2003.223.1293 14621216

[r403] Hsu Y and Sun L 2010 Factors associated with aggressive responses in pet dogs. Applied Animal Behaviour Science 123(3–4), 108–123. 10.1016/j.applanim.2010.01.013

[r70] ICECDogs 2024 *International Collaborative on Extreme Conformation in Dogs.* https://www.icecdogs.com/ (accessed 14 March 2024).

[r71] IEWG 2024 *International Elbow Working Group.* http://www.vet-iewg.org/ (accessed 8 August 2024).

[r72] Ikolo F, Maity S, Finn R, Abdullah A, Tajic A, Cameron JM, Maj MC, Ikolo F, Maity S, Finn R, Abdullah A, Tajic A, Cameron JM and Maj MC 2023 *Founder Effect: Breeding a Dog for the Elderly Gentleman Reveals an Animal Model of a Human Genetic Disorder.* IntechOpen. 10.5772/intechopen.113912

[r73] IPFD 2024 *International Partnership For Dogs.* https://dogwellnet.com/ctp/ (accessed 8 August 2024).

[r74] Irish Red and White Setter Club 2024 *OutCross Program.* https://www.irishredandwhitesetterclub.org/outcross-program (accessed 23 January 2024).

[r75] Janssens L, Giemsch L, Schmitz R, Street M, Van Dongen S and Crombé P 2018 A new look at an old dog: Bonn-Oberkassel reconsidered. Journal of Archaeological Science 92: 126–138. 10.1016/j.jas.2018.01.004

[r76] Jensen VF and Christensen KA 2000 Inheritance of disc calcification in the Dachshund. Journal of Veterinary Medicine Series A 47(6): 331–340. 10.1046/j.1439-0442.2000.00297.x 11008442

[r77] Jordbruksverket 2001 *Märk och registrera hundar.* https://jordbruksverket.se/djur/hundar-katter-och-smadjur/hundar/mark-och-registrera-hundar (accessed 25 January 2024). [Title translation: Tag and register dogs].

[r78] Jung C and Pörtl D 2018 Scavenging hypothesis: Lack of evidence for dog domestication on the waste dump. Dog Behaviour 4(2). 10.4454/db.v4i2.73

[r79] KC 2019 *Britain’s top dogs: Old favourites rise & fall | Kennel Club.* https://www.thekennelclub.org.uk/media-centre/2019/november/britains-top-dogs-old-favourites-rise-and-fall-while-continental-cousins-skyrocket/ (accessed 13 December 2023).

[r80] KC 2023 *The Kennel Club standard, Bulldog.* https://www.thekennelclub.org.uk/breed-standards/utility/bulldog/ (accessed 4 April 2024).

[r81] KC 2024 *Breed registration statistics in the Kennel Club.* https://www.thekennelclub.org.uk/media-centre/breed-registration-statistics/ (accessed 24 January 2024).

[r82] Kiviranta AM, Rusbridge C, Laitinen-Vapaavuori O, Hielm-Björkman A, Lappalainen AK, Knowler SP and Jokinen TS 2017 Syringomyelia and craniocervical junction abnormalities in Chihuahuas. Journal of Veterinary Internal Medicine 31: 1771–1781. 10.1111/jvim.14826 28892202 PMC5697179

[r83] Knap PW and Su G 2008 Genotype by environment interaction for litter size in pigs as quantified by reaction norms analysis. Animal 2(12): 1742–1747. 10.1017/S1751731108003145 22444079

[r84] Kraus C, Snyder-Mackler N and Promislow DEL (2023) How size and genetic diversity shape lifespan across breeds of purebred dogs. GeroScience 45(2), 627–643. 10.1007/s11357-022-00653-w 36066765 PMC9886701

[r85] LAGECDogs 2024a *Legal Advisory Group on Extreme Conformation in Dogs.* https://www.alaw.org.uk/companion-animals/extreme-dog-conformation/ (accessed 24 January 2024).

[r86] LAGECDogs 2024b *Penalties for breeding dogs in breach of licensing requirements in England.* https://www.alaw.org.uk/wp-content/uploads/2024/04/LAGECDogs-Penalties-for-breeding-dogs-in-breach-of-licencing-requirements-March-2024.pdf (accessed 13 November 2024).

[r87] Lawler DF 2012 Pedigree Dog “Exposed”: A Documentary. Journal of Applied Animal Welfare Science 15(2): 181–185. 10.1080/10888705.2012.658335 22458877

[r88] Leeb T 2023 Special issue: “Canine genetics 2”. Genes 14(10): 1930. 10.3390/genes14101930 37895280 PMC10606197

[r89] Legislation 2006 *Animal Welfare Act.* https://www.legislation.gov.uk/ukpga/2006/45/contents (accessed 30 January 2024).

[r90] Legislation 2018 *Licensing of Activities Involving Animals Regulations.* https://www.legislation.gov.uk/uksi/2018/486/contents/made (accessed 9 February 2024).

[r91] Lemonick MD 2001 *A Terrible Beauty.* https://web.archive.org/web/20060820094419/http://www.time.com/time/magazine/article/0,9171,163404,00.html (accessed 24 January 2024).

[r92] Leroy G and Baumung R 2011 Mating practices and the dissemination of genetic disorders in domestic animals, based on the example of dog breeding. Animal Genetics 42(1): 66–74. 10.1111/j.1365-2052.2010.02079.x 20528847

[r93] Lewis TW, Wiles BM, Llewellyn-Zaidi AM, Evans KM and O’Neill DG 2018 Longevity and mortality in Kennel Club registered dog breeds in the UK in 2014. Canine Genetics and Epidemiology 5(1): 10. 10.1186/s40575-018-0066-8 30349728 PMC6191922

[r94] Lewis TW, Woolliams JA and Blott SC 2010 Optimisation of breeding strategies to reduce the prevalence of inherited disease in pedigree dogs. Animal Welfare 19(S1): 93–98. 10.1017/S0962728600002281

[r95] Liu N-C, Troconis EL, Kalmar L, Price DJ, Wright HE, Adams VJ, Sargan DR and Ladlow JF 2017 Conformational risk factors of brachycephalic obstructive airway syndrome (BOAS) in pugs, French bulldogs, and bulldogs. PLoS One 12(8): e0181928. 10.1371/journal.pone.0181928 28763490 PMC5538678

[r96] Lovdata 2009 *Lov om dyrevelferd - Lovdata.* https://lovdata.no/dokument/LTI/lov/2009-06-19-97 (accessed 13 December 2023). [Title translation: Animal welfare act].

[r97] MacLean EL, Snyder-Mackler N, von Holdt BM and Serpell JA 2019 Highly heritable and functionally relevant breed differences in dog behaviour. Proceedings of the Royal Society B: Biological Sciences 286(1912): 20190716. 10.1098/rspb.2019.0716 PMC6790757 31575369

[r98] Mäki K 2023 *Stop breeding, combine all breeds - would these means reduce the harms of inbreeding in pedigree dogs?* https://www.katariinamaki.fi/l/reducing-the-harms-of-inbreeding-in-pedigree-dogs-by-combining-all-dog-breeds/ (accessed 15 May 2024).

[r99] Markarian T 2023 What the judge wants to see. *Reader’s Digest.* https://www.rd.com/article/why-beloved-dog-breeds-will-never-win-westminster/ (accessed 2 April 2024).

[r100] Marsden CD, Ortega-Del Vecchyo D, O’Brien DP, Taylor JF, Ramirez O, Vilà C, Marques-Bonet T, Schnabel RD, Wayne RK and Lohmueller KE 2016 Bottlenecks and selective sweeps during domestication have increased deleterious genetic variation in dogs. Proceedings of the National Academy of Sciences 113(1): 152–157. 10.1073/pnas.1512501113 PMC4711855 26699508

[r101] McGreevy PD 2007 Breeding for quality of life. Animal Welfare 16(S): 125–128. 10.1017/S0962728600031821

[r102] McGreevy PD and Nicholas FW 1999 Some practical solutions to welfare problems in dog breeding. Animal Welfare 8(4): 329–341. 10.1017/S0962728600021965

[r103] McMillan KM, Bielby J, Williams CL, Upjohn MM, Casey RA and Christley RM 2024 Longevity of companion dog breeds: those at risk from early death. Scientific Reports 14(1): 531. 10.1038/s41598-023-50458-w 38302530 PMC10834484

[r104] Melis C, Pertoldi C, Ludington WB, Beuchat C, Qvigstad G and Stronen AV 2022 Genetic Rescue of the Highly Inbred Norwegian Lundehund. Genes 13(1), 163. 10.3390/genes13010163 35052503 PMC8775414

[r105] Meuwissen T, Hayes B and Goddard M 2016 Genomic selection: A paradigm shift in animal breeding. Animal Frontiers 6(1): 6–14. 10.2527/af.2016-0002

[r106] Morey DF 2006 Burying key evidence: the social bond between dogs and people. Journal of Archaeological Science 33(2): 158–175. 10.1016/j.jas.2005.07.009

[r107] Morrill K, Hekman J, Li X, McClure J, Logan B, Goodman L, Gao M, Dong Y, Alonso M, Carmichael E, Snyder-Mackler N, Alonso J, Noh HJ, Johnson J, Koltookian M, Lieu C, Megquier K, Swofford R, Turner-Maier J, White ME, Weng Z, Colubri A, Genereux DP, Lord KA and Karlsson EK 2022 Ancestry-inclusive dog genomics challenges popular breed stereotypes. Science 376(6592): eabk0639. 10.1126/science.abk0639 35482869 PMC9675396

[r108] Nicholas CE, Wegienka GR, Havstad SL, Zoratti EM, Ownby DR and Johnson CC 2011 Dog allergen levels in homes with hypoallergenic compared with nonhypoallergenic dogs. American Journal of Rhinology & Allergy 25(4): 252–256. 10.2500/ajra.2011.25.3606 21819763 PMC3680143

[r109] Nicholas FW 2010 Introduction to Veterinary Genetics, *Third Edition.* John Wiley & Sons: London, UK.

[r110] Nicholas FW 2011 Response to the documentary Pedigree Dogs Exposed: Three reports and their recommendations. The Veterinary Journal 189(2): 126–128. 10.1016/j.tvjl.2011.06.007 21742520

[r111] Nicholas FW, Arnott ER and McGreevy PD 2016 Hybrid vigour in dogs? The Veterinary Journal 214: 77–83. 10.1016/j.tvjl.2016.05.013 27387730

[r112] NKU 2023 *Breed Specific Instructions.* https://www.skk.se/en/nku-home/projects/breed-specific-instructions/ (accessed 24 January 2024).

[r113] O’Brien MJ, Beijerink NJ and Wade CM 2021 Genetics of canine myxomatous mitral valve disease. Animal Genetics 52(4): 409–421. 10.1111/age.13082 34028063

[r114] Oliehoek PA, Bijma P and van der Meijden A 2009 History and structure of the closed pedigree population of Icelandic Sheepdogs. Genetics Selection Evolution 41(1): 39. 10.1186/1297-9686-41-39 PMC2736928 19660133

[r115] O’Neill DG, Church DB, McGreevy PD, Thomson PC and Brodbelt DC 2013 Longevity and mortality of owned dogs in England. The Veterinary Journal 198(3): 638–643. 10.1016/j.tvjl.2013.09.020 24206631

[r116] O′Neill DG, Church DB, McGreevy PD, Thomson PC and Brodbelt DC 2014 Prevalence of disorders recorded in dogs attending primary-care veterinary practices in England. PLoS One 9(3): e90501. 10.1371/journal.pone.0090501 24594665 PMC3942437

[r117] O’Neill DG, McMillan KM, Church DB and Brodbelt DC 2023 Dog breeds and conformations in the UK in 2019: VetCompass canine demography and some consequent welfare implications. PLoS One 18(7): e0288081. 10.1371/journal.pone.0288081 37494312 PMC10370710

[r118] O’Neill DG, Pegram C, Crocker P, Brodbelt DC, Church DB and Packer RMA 2020 Unravelling the health status of brachycephalic dogs in the UK using multivariable analysis. Scientific Reports 10(1): 17251. 10.1038/s41598-020-73088-y 33057051 PMC7560694

[r119] O’Neill DG, Rowe D, Brodbelt DC, Pegram C and Hendricks A 2022a Ironing out the wrinkles and folds in the epidemiology of skin fold dermatitis in dog breeds in the UK. Scientific Reports 12(1): 10553. 10.1038/s41598-022-14483-5 35794173 PMC9259571

[r120] O’Neill DG, Sahota J, Brodbelt DC, Church DB, Packer RMA and Pegram C 2022b Health of Pug dogs in the UK: disorder predispositions and protections. Canine Medicine and Genetics 9(1): 4. 10.1186/s40575-022-00117-6 35581668 PMC9115981

[r121] Ostrander EA and Wayne RK 2005 The canine genome. Genome Research 15(12): 1706–1716. 10.1101/gr.3736605 16339369

[r122] Overheid 2024 *Besluit houders van dieren.* https://wetten.overheid.nl/BWBR0035217/2018-07-01 (accessed 9 February 2024). [Title translation: Animal keepers decree].

[r123] Packer R and O’Neill D 2022 Health and Welfare of Brachycephalic (Flat-Faced) Companion Animals: A Complete Guide for Veterinary and Animal Professionals, First Edition. CRC Press: Boca Raton, FL, USA.

[r124] Packer RM, Hendricks A, Tivers MS and Burn CC 2015 Impact of facial conformation on canine health: brachycephalic obstructive airway syndrome. PLoS One 10(10): e0137496. 10.1371/journal.pone.0137496 26509577 PMC4624979

[r125] Parker HG, Kim LV, Sutter NB, Carlson S, Lorentzen TD, Malek TB, Johnson GS, DeFrance HB, Ostrander EA and Kruglyak L 2004 Genetic structure of the purebred domestic domestic dog. Science 304(5674): 1160–1164. 10.1126/science.1097406 15155949

[r126] Pegram C, Raffan E, White E, Ashworth AH, Brodbelt DC, Church DB and O’Neill DG 2021 Frequency, breed predisposition and demographic risk factors for overweight status in dogs in the UK. Journal of Small Animal Practice 62(7): 521–530. 10.1111/jsap.13325 33754373

[r127] Peyer N and Steiger A 1998 The assessment of breed defects in dogs in relation to animal welfare. Schweizer Archiv fur Tierheilkunde 140(9): 359–364. 9757783

[r128] Powell, D 2011 *The Dalmatian/Pointer Backcross Project.* https://www.vin.com/doc/?id=6698829 (accessed 23 January 2024)

[r129] Powell L, Chia D, McGreevy P, Podberscek AL, Edwards KM, Neilly B, Guastella AJ, Lee V and Stamatakis E 2018 Expectations for dog ownership: Perceived physical, mental and psychosocial health consequences among prospective adopters. PLoS One 13(7): e0200276. 10.1371/journal.pone.0200276 29979749 PMC6034856

[r130] Power ER 2012 Domestication and the dog: embodying home. Area 44(3): 371–378. 10.1111/j.1475-4762.2012.01098.x

[r131] Proschowsky HF, Rugbjerg H and Ersbøll AK 2003 Mortality of purebred and mixed-breed dogs in Denmark. Preventive Veterinary Medicine 58(1–2): 63–74. 10.1016/S0167-5877(03)00010-2 12628771

[r132] Raad van Beheer 2024 *DNA ancestry check.* https://www.houdenvanhonden.nl/ (accessed 23 April 2024).

[r133] Redmalm D 2014 Holy bonsai wolves: Chihuahuas and the Paris Hilton syndrome. International Journal of Cultural Studies 17(1): 93–109. 10.1177/1367877912464539

[r134] Retsinformation 2018 *BEK nr 1044 af 10/08/2018.* Ministeriet for Fødevarer, Landbrug og Fiskeri. http://www.retsinformation.dk/eli/lta/2018/1044 (accessed 12 December 2023).

[r135] Ritvo H 1987 The Animal Estate: The English and Other Creatures in the Victorian Age. Harvard University Press: Cambridge, MA, USA.

[r136] Rodenburg T, Buitenhuis A, Ask B, Uitdehaag K, Koene P, Van Der Poel J and Bovenhuis H 2003 Heritability of feather pecking and open-field response of laying hens at two different ages. Poultry Science 82(6): 861–867. 10.1093/ps/82.6.861 12817438

[r137] Saetre P, Strandberg E, Sundgren P-E, Pettersson U, Jazin E and Bergström TF 2006 The genetic contribution to canine personality. Genes, Brain and Behaviour 5(3): 240–248. 10.1111/j.1601-183X.2005.00155.x 16594977

[r138] Salonen M, Mikkola S, Niskanen JE, Hakanen E, Sulkama S, Puurunen J and Lohi H 2023 Breed, age, and social environment are associated with personality traits in dogs. iScience 6(5): 106691. 10.1016/j.isci.2023.106691 PMC10165416 37168553

[r139] Sampson J and Binns M 2006 The Kennel Club and the early history of dog shows and breed clubs. Cold Spring Harbor Monograph Series 44: 19.

[r140] Sandøe P, Corr S and Palmer C 2015 Companion Animal Ethics. John Wiley & Sons: London, UK.

[r141] Sandøe P, Jensen J, Jensen F and Nielsen S 2019 Shelters reflect but cannot solve underlying problems with relinquished and stray animals—a retrospective study of dogs and cats entering and leaving shelters in Denmark from 2004 to 2017. Animals 9(10): 765. 10.3390/ani9100765 31590389 PMC6826399

[r142] Sandøe P, Lund TB, Nielsen SS, Proschowsky HF, Ralund S and Romberg P 2022 Hvor kommer danske hunde fra? *Dansk Veterinærtidsskrift.* https://dvt.ddd.dk/artikler/artikler/hvor-kommer-danske-hunde-fra/ (accessed 3 April 2024). [Title translation: Where do Danish dogs come from?].

[r143] Schofield I, Woolhead V, Johnson A, Brodbelt DC, Church DB and O’Neill DG 2021 Hypoadrenocorticism in dogs under UK primary veterinary care: frequency, clinical approaches and risk factors. Journal of Small Animal Practice 62(5): 343–350. 10.1111/jsap.13285 33555046 PMC8248152

[r144] Serpell J 2017 The Domestic Dog. Cambridge University Press: Cambridge, UK.

[r145] Serpell J 2019 How happy is your pet? The problem of subjectivity in the assessment of companion animal welfare. Animal Welfare 28(1): 57–66. 10.7120/09627286.28.1.057

[r146] Serpell J 2021 Commensalism or cross-species adoption? A critical review of theories of wolf domestication. Frontiers in Veterinary Science 8: 662370. 10.3389/fvets.2021.662370 33937382 PMC8083978

[r147] Shouldice VL, Edwards AM, Serpell JA, Niel L and Robinson JAB 2019 Expression of behavioural traits in Goldendoodles and Labradoodles. Animals 9(12): 1162. 10.3390/ani9121162 31861203 PMC6940824

[r148] Shrader SM, Jung S, Denney TS and Smith BF 2018 Characterization of Australian labradoodle dystrophinopathy. Neuromuscular Disorders 28(11): 927–937. 10.1016/j.nmd.2018.08.008 30286978

[r149] SKC 2023 *Ja till inkorsningsprojekt för cavalier.* https://www.skk.se/nyheter/2023/06/ja-till-inkorsningsprojekt-for-cavalier/ (accessed 15 May 2024). [Title translation: Yes to crossbreeding project for cavalier].

[r150] Skipper A 2020 The ‘dog doctors’ of Edwardian London: Elite canine veterinary care in the early Twentieth Century. Social History of Medicine 33(4): 1233–1258. 10.1093/shm/hkz049 33469408 PMC7805801

[r151] Skipper A 2022a A historical perspective on brachycephalic breed health and the role of the veterinary profession. In: Packer RMA and O’Neill DG (eds) Health and Welfare of Brachycephalic (Flat-faced) Companion Animals pp 7–24. CRC Press/Taylor and Francis: Abingdon, UK.

[r152] Skipper A 2022b Form, function and fashion: health, disease and British pedigree dog breeding during the long twentieth century. Doctoral Thesis, King’s College London, UK.

[r153] Supreme court of Norway 2023 *Breeding of Cavalier King Charles Spaniels is a violation the Animal Welfare Act, while breeding of English Bulldogs under a certain breeding program is not.* https://www.domstol.no/en/supremecourt/rulings/2023/supreme-court-civil-cases/HR-2023-1901-A/ (accessed 13 December 2023).

[r154] Sutter NB, Bustamante CD, Chase K, Gray MM, Zhao K, Zhu L, Padhukasahasram B, Karlins E, Davis S, Jones PG, Quignon P, Johnson GS, Parker HG, Fretwell N, Mosher DS, Lawler DF, Satyaraj E, Nordborg M, Lark KG, Wayne RK and Ostrander EA 2007 A single *IGF1* allele is a major determinant of small size in dogs. Science 316(5821): 112–115. 10.1126/science.1137045 17412960 PMC2789551

[r155] Swiss Cynological Federation 2024 *Der Labradoodle.* https://www.srz-schweiz.org/der-labradoodle?lang=en (accessed 9 February 2024).

[r156] TASSO 2023 *TASSO e.V. Haustierregister.* https://www.tasso.net (accessed 25 January 2024).

[r157] Teng KT, Brodbelt DC, Pegram C, Church DB and O’Neill DG 2022 Life tables of annual life expectancy and mortality for companion dogs in the United Kingdom. Scientific Reports 12(1): 6415. 10.1038/s41598-022-10341-6 35484374 PMC9050668

[r158] The Kennel Club 2024 *Breeds A to Z | The Kennel Club.* https://www.thekennelclub.org.uk/search/breeds-a-to-z/ (accessed 14 May 2024).

[r159] Tinbergen N 1951 The Study of Instinct. Oxford University Press: Oxford, UK, Clarendon Press: New York, USA.

[r160] Turcsán B, Miklósi Á and Kubinyi E 2017 Owner perceived differences between mixed-breed and purebred dogs. PLoS One 12(2): e0172720. 10.1371/journal.pone.0172720 28222103 PMC5319786

[r161] van Hagen M 2020 *Criteria for breeding healthy short-nosed dogs.* https://www.uu.nl/en/background/criteria-for-breeding-healthy-short-nosed-dogs (accessed 30 January 2024).

[r162] VetCompass 2024 *VetCompass.* Royal Veterinary College. https://www.rvc.ac.uk/VetCOMPASS (accessed 29 January 2024).

[r163] Vredegoor DW, Willemse T, Chapman MD, Heederik DJJ and Krop EJM 2012 Can f 1 levels in hair and homes of different dog breeds: Lack of evidence to describe any dog breed as hypoallergenic. Journal of Allergy and Clinical Immunology 130(4): 904–909. 10.1016/j.jaci.2012.05.013 22728082

[r164] Walsh JH 1879 The Dog in Health and Disease, *Third Edition.* Longmans, Green, and Co: London, UK.

[r165] Wisdom Panel 2024 *Wisdom Panel breed discovery.* https://www.wisdompanel.com/en-us/dog-dna-tests/breed-discovery (accessed 23 January 2024).

[r166] Worboys M, Strange J-M and Pemberton N 2018 The Invention of the Modern Dog: Breed and Blood in Victorian Britain. JHU Press: Baltimore, USA.

[r167] Zangerl B, Goldstein O, Philp AR, Lindauer SJ, Pearce-Kelling SE, Mullins RF, Graphodatsky AS, Ripoll D, Felix JS and Stone EM 2006 Identical mutation in a novel retinal gene causes progressive rod–cone degeneration in dogs and retinitis pigmentosa in humans. Genomics 88(5): 551–563. 10.1016/j.ygeno.2006.07.007 16938425 PMC3989879

